# Intermittent Fasting for the Prevention of Cardiovascular Disease Risks: Systematic Review and Network Meta-Analysis

**DOI:** 10.1007/s13668-025-00684-7

**Published:** 2025-07-24

**Authors:** Kelemu Tilahun Kibret, Anna Peeters, Teketo Kassaw Tegegne, Yonatan Moges Mesfin, Melanie Nichols

**Affiliations:** 1 Global Centre for Preventive Health and Nutrition, Institute for Health Transformation, School of Health and Social Development, Faculty of Health, Deakin University, Geelong, VIC 3220 Australia; 2 Institute for Physical Activity and Nutrition, Deakin University, Geelong, VIC 3220 Australia; 3 Murdoch Children’s Research Institute, Asian-Pacific Health, Immunity and Global Health, Infection, Parkville, VIC 3052 Australia; 4 Victorian Health Promotion Foundation (VicHealth), 355 Spencer St, West Melbourne, VIC 3003 Australia

**Keywords:** Intermittent fasting, Cardiometabolic risk factors, Network meta-analysis

## Abstract

**Context:**

While several studies have assessed the potential effect of intermittent fasting on reducing cardiovascular risks, the findings are inconclusive.

**Objective:**

To compare the relative effectiveness of intermittent fasting methods in reducing key cardiovascular risks.

**Methods:**

Studies were searched from Medline, Embase, Cochrane Library Central and Global Health to identify studies that enrolled adults (≥ 18 years) to intermittent fasting methods and reported effects on one of the six specified cardiovascular risk factors. We performed a random-effects network meta-analysis using a frequentist framework. Outcomes were reported as mean differences (MD) with their corresponding 95% confidence intervals (CI).

**Results:**

Fifty-six studies were included in the analysis. With high certainty of evidence, modified alternate-day fasting was found to be the most effective intervention compared to a usual diet in reducing body weight (MD= -5.18 kg; 95% CI: -7.04, -3.32), waist circumference (-3.55 cm; -5.66, -1.45), systolic blood pressure (-7.24 mmHg; -11.90, -2.58), diastolic blood pressure (-4.70 mmHg; -8.46, -0.95). With high certainty, time-restricted eating was the most effective intervention compared to usual diet in reducing fat-free mass (-0.82 kg; -1.46, -0.17), waist circumference (-3.00 cm; -4.50, -1.51), diastolic blood pressure (-3.24 mmHg; -4.69, -1.79) and fasting plasma glucose (-3.74 mg/dL; -6.01, -1.46).

**Conclusions:**

Modified alternate-day fasting, and time-restricted eating appear to be promising approaches for reducing most cardiovascular risk factors. These intermittent fasting methods may be considered as potential components of lifestyle interventions aimed at managing cardiovascular disease risk factors. However, further long-term randomised controlled trials comparing intermittent fasting methods are needed to confirm their efficacy and assess their safety over time.

**Supplementary Information:**

The online version contains supplementary material available at 10.1007/s13668-025-00684-7.

## Introduction

Cardiovascular diseases (CVD) are a leading cause of morbidity and mortality worldwide, affecting individuals in high-income as well as low-and middle-income countries [1]. The main contributors to the major cardiovascular diseases (ischemic heart disease and stroke) include overweight or obesity, high blood pressure, high blood glucose, and dyslipidaemia [1, 2]. Behavioural modification including dietary intake and physical activity is an important approach to mitigate cardiometabolic risk factors such as overweight or obesity, high blood pressure, elevated cholesterol levels and blood glucose [3, 4]. Weight control through energy restriction has been shown to improve cardiovascular risks including insulin resistance, blood glucose, and blood pressure [5].

Intermittent fasting, which includes a range of approaches to achieve overall energy restriction, has emerged as an appealing alternative to continuous energy restriction (CER) for managing obesity and its related comorbidities due to its relative ease of maintaining long-term adherence [6, 7]. Intermittent fasting refers to dietary patterns that involve cycling between periods of eating and periods of fasting [8]. This creates periods of energy deficit, and metabolic change which can potentially leading to health benefits, including weight loss, improved insulin sensitivity, and better overall metabolic health [6, 9].

Among the many methods of intermittent fasting, some of the most adopted include alternate-day fasting (ADF), modified alternate day fasting (mADF), periodic fasting (PF), and time-restricted eating (TRE) [6, 10]. ADF is cyclic eating approach involves a 24-hour period of complete fasting (no calorie intake) followed by a 24-hour period of normal eating [8, 11]. The mADF is like ADF but allows for some calorie intake on fasting days (25% or less intake of energy) [8, 11]. PF is a cyclical weekly eating pattern with fasting for one or two days per week (consumption of 25% or less of required calories or restricting calorie intake to around 500–600 kcal/day) and then eating normally for the remaining five or six days a week. The 5:2 diet is a popular form of PF [8, 12].TRE involves complete fasting (no calorie intake) for at least 12 h per day and eating freely the rest of the time [8, 12]. TRE involves limiting the daily eating window to a specific period, for example, an individual might eat all meals within an 8-hour window (e.g., 12:00 pm to 8:00 pm) and fast for the remaining 16 h each day (16/8 method). The most common TRE methods are the 16/8 and 14/10 method [8, 12].

Previous pairwise meta-analysis studies have shown some promise for intermittent fasting in reducing risk factors for cardiovascular disease. However, the results are not consistent [8, 11, 13]. Some meta-analyses suggest that intermittent fasting is more effective than usual eating pattern in reducing weight and waist circumference [11–14]. However, others showed no significant difference between intermittent fasting and CER for these measures [15, 16]. Regarding fat-free mass, there is no clear conclusion on whether intermittent fasting leads to undesirable loss of muscle mass. Some studies found no effect [13, 16], while others showed an increase [17] or decrease [15] compared to usual diet. Findings on blood pressure are also inconsistent. Some meta-analyses suggest intermittent fasting reduces systolic blood pressure (SBP) and diastolic blood pressure (DBP) compared to usual eating [11, 12], while others found no significant difference [13, 14]. Similarly, some studies showed intermittent fasting reduced fasting blood sugar [11, 14] and low-density lipoprotein (LDL) cholesterol [18], but others found no significant difference compared to usual eating on fasting plasma glucose (FPG) [13] and LDL reduction [11, 12, 14]. The inconsistencies of results across the previous meta-analyses could be due to differences in terms of the population, the intervention duration (some included short duration studies) [12–14] and number of studies included [12–14]. Further, some conducted the analyses by combining all intermittent fasting methods together [11, 12, 16].

Since conventional pairwise meta-analysis is often limited by comparing two intervention at a time and cannot incorporate indirect evidence, there remains considerable uncertainty about which intermittent fasting methods are the most effective for improving cardiovascular health [19]. An alternative approach is network meta-analysis (NMA) which allows statistical comparison of three or more interventions that have not been directly compared in randomised controlled trials (RCTs) (19). Furthermore, NMA has the potential to enhance the precision of effect estimates derived from RCTs and traditional pairwise meta-analyses by integrating both direct and indirect evidence (19). This method offers a more thorough understanding of relative effectiveness and allows for the ranking of intermittent fasting methods, which is not possible with conventional pairwise meta-analysis. The aim of this systematic review and network meta-analysis was to assess the relative effectiveness of different intermittent fasting methods in improving key cardiovascular risk factors, including body weight, waist circumference, fat free mass, elevated blood pressure, FPG, low density lipoprotein cholesterol.

## Methods

The protocol was registered at PROSPERO (CRD42023475279), and the NMA was reported in accordance with the Preferred Reporting Items for Systematic Review and Meta-analysis for Network Meta-Analyses (PRISMA-NMA) guidelines [20] (see supplementary material (S1).

### Search Strategy

We searched four databases: Medline, Embase, Cochrane Library, and Global Health—from inception to November 09, 2023, and the search was updated up to December 11, 2024. We also performed manual searches of references from relevant reviews and eligible studies. The key search terms include a combination of “intermittent fasting” or “alternate day fasting” or “periodic fasting” or “time restricted eating /feeding” or “intermittent energy restriction” and body weight or waist circumference or fat-free mass or blood pressure or SBP or DBP or LDL or fasting plasma/blood glucose. The full search strategy is presented in the supplementary material (S2). The search was limited to RCTs, published in English. There was no limitation on publication date or location. Search results were exported to Covidence for duplicate removal, screening and data extraction.

### Eligibility Criteria

We developed the eligibility criteria based on the PICOS framework (Participants, Interventions, Comparisons, Outcomes, and Study design). All inclusion and exclusion criteria are summarised in Table 1. This systematic review and network meta-analysis included only RCTs.

**Table 1:** PICOS criteria for inclusion and exclusion of studies

Parameter	Inclusion Criteria	Exclusion criteria
Participant/ Population	Adults aged ≥ 18 years with or without cardiometabolic risk factors but without other chronic diseases such as cancer, non-alcoholic fatty liver disease	studies conducted on children, pregnant women, or animals;
Interventions	One or more types of intermittent fasting (ADF, mADF, PF, TRE) lasting at least for two weeks	studies that combined intermittent fasting with other interventions, such as intermittent fasting plus Mediterranean diets or exercise
Comparators	At least one comparator arm, which could be either control group without intervention (unchanged eating habit or on usual diet) or another intermittent fasting type.	If comparison is one intermittent fasting based on time (e.g. ealy TRE vs. late TRE)
Outcomes	RCTs reporting effect sizes or changes in data before and after the intervention were included if they assessed at least one of the following cardiometabolic risks: body weight, waist circumference, fat-free mass, blood pressure (SBP and DBP), fasting blood glucose, or LDL cholesterol	studies that did not include any of the outcomes of interest or did not present sufficient information
Study designs	RCTs conducted in developed or developing countries	Religious fasting studies, pre-post studies, studies with small sample size (*n* < 10); non-randomised controlled trails, including cohort studies, case-control studies, cross-sectional studies, reviews, case reports, and conference abstracts.

### Screening and Data Extraction

**Fig. 1:**
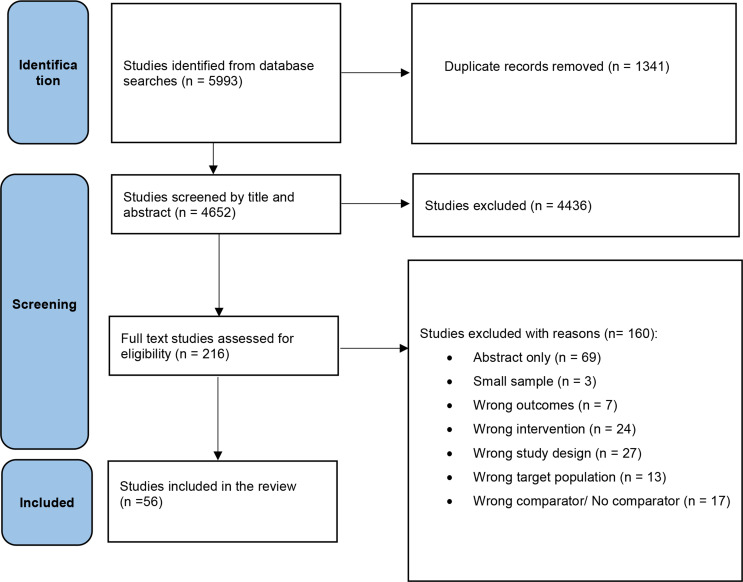
The PRISMA study selection flow diagram

Three independent reviewers conducted the title and abstract screening: KTK screened all, TKT screened 69%, and YMM screened 31%. KTK performed the full-text review, with TKT double-checking 20%, applying the inclusion and exclusion criteria. Any discrepancies between the reviewers were resolved through discussion and consensus. The step-by-step procedure of identifying, screening, and incorporating or excluding studies presented using the PRISMA 2020 flow diagram (Fig. 1). Data were extracted using a pretested data abstraction form. The following information was extracted from each eligible study: first author, publication year, country, the intervention duration, sample size, participant characteristics (sex, age, BMI) and outcomes measured, intervention or intermittent fasting type (s), control group diet, number of participants in each group (treatment and control group). If intermittent fasting outcomes were reported at multiple time points, we extracted data from the last reported time point or the end of the intervention.

For studies reporting pre- and post-intervention measures, we calculated mean differences and standard deviations using Cochrane Handbook methods [21]. Missing standard deviations were estimated from standard errors or confidence intervals. For studies that reported only medians and interquartile ranges, means were estimated using the Wan method [22]. In cases the data were only available in figures, numerical data was obtained using Plot Digitis*er* (https://plotdigitizer.com/app).

### Risk of Bias Assessment

We assessed the risk of bias using the Cochrane Collaboration’s Risk of Bias 2 (Rob 2) tool for RCTs [23]. This tool comprises five bias components: bias in the randomization process, bias resulting from deviations in intended interventions, bias due to missing outcome data, bias in the measurement of outcomes, and bias in the selection of reported results. Each study was assessed and categorised according to its risk of bias into three levels (low risk of bias, some concerns, or high risk of bias), for each domain evaluated. A study was deemed to have a low overall risk of bias if all domains were rated as having a low risk of bias. Conversely, a study was considered to have a high risk of bias if at least one domain is rated as high risk, or if three and more domains were categorised as having ‘some concerns’. A study would fall into the ‘some concerns’ category overall if one or two of the domains are rated as having some concerns, but none were classified as high risk of bias [23].

### Grading the Certainty of Evidence

We assessed the certainty of the evidence using Grading of Recommendation Assessment, Development, and Evaluation (GRADE) approach [24]. We classified the certainty of evidence as high, moderate, low, or very low. RCTs initially receive a high grade; however, this grade may be downgraded due to the following specific criteria: the presence of risk of bias (weight assigned to study as assessed by the RoB2 tool); inconsistency (significant unexplained variation among study results, indicated by I^2^), indirectness (limitations in the generalizability of the results); imprecision (wide 95% confidence intervals for effect estimates or crossing a null value); incoherence (differences between direct and indirect estimates that contribute to a network estimate); and publication bias (significant evidence of small-study effects) [24–26].

### Statistical Analysis

We performed a random-effects network meta-analysis using a frequentist framework to the compare the effectiveness of different intermittent fasting methods on cardiovascular disease risks. We chose the frequentist approach over a Bayesian framework for its computational efficiency and straightforward implementation using standard statistical software. Additionally, the frequentist method provides robust and interpretable estimates without requiring prior distributions, which were not available for all comparisons in our network. We reported outcomes as mean differences (MD) with their 95% confidence intervals (CI). We created the network geometry diagrams to explore networks of intervention comparisons. The size of the nodes, representing each intervention, reflects total number of participants while the thickness of the lines connecting any two nodes illustrates the number of intervention comparisons. The incoherence assumption was checked by using a statistical test (network node-splitting method). In a closed-loop network, the node-splitting method was used to test incoherence between direct and indirect intervention comparisons [27]. We assessed incoherence by comparing the similarity of point estimates, checking for overlapping 95% confidence intervals, and ensuring non-significant p-values.

Transitivity was ensured by including only RCTs with comparable populations, interventions, and outcomes, and verifying that all included studies could be meaningfully compared based on shared treatment nodes. Multilevel meta-analysis was not conducted due to the primary focus on treatment comparisons across studies rather than variability within individual trials.

The relative rankings of all intermittent fasting methods for each outcome were determined by estimating ranking probabilities using ranking plots and the surface under the cumulative ranking curve (SUCRA) [28, 29].

### Classification of Intermittent Fasting Methods as More and Less Effective Intervention

Using a new GRADE approach, we analysed NMA results by classifying intermittent fasting interventions from the most to least effective [30] for each outcome. The new GRDAE approach considers three factors: effect size from the NMA, evidence certainty, and SUCRA (ranking) values [30]. We first categorised evidence quality into high (moderate-to-high) and low (low-to-very-low) certainty. Within each category, intermittent fasting method were divided based on their effect on outcomes: (1) **Most Effective**: intermittent fasting method with the largest reduction in outcomes compared to the usual diet and superior to at least one moderately effective method; **(2) Moderately Effective**: intermittent fasting method better than the usual diet but not as effective as the most effective method; (3) **Least Effective**: intermittent fasting method similar to the usual diet, with confidence intervals crossing zero.

### Sensitivity Analysis

We conducted sensitivity analysis to assess the stability or robustness of the pooled effect size by restricting the analysis to studies with medium to long-term intervention durations, some concern or low risk of bias, and studies that did not include participants with diabetes.

Data analysis was conducted using Stata version 18.0 (StataCorp, College Station, TX) [31], and all graphical displays were generated using the tools developed by Chaimani et al. and White [31, 32].

## Results

### Study Selection and Characteristics

A total of 5993 articles were identified, resulting in the inclusion of 56 studies [33–88] (Fig. 1). These 56 studies were conducted between 2013 and 2024 with a sample size ranging from 18 to 222 and totalling 3,965 participants. The studies were carried out in 16 different countries, including the USA (*n* = 17), Australia (*n* = 8), China (*n* = 6), and Norway (*n* = 4). The duration of interventions varied from 4 weeks to 104 weeks. Of the 56 studies, seven were three-arm while the rest were two-arm studies. The mean age of participants was 45.0 (SD 10.1) years (see details in Table 2).

**Table 2:** Characteristics of included stidies

Study ID	Country	Total participants	Study population description	Interventions groups	Sample	Mean age	Male	Female	BMI	Intervention detail	Intervention duration (wks)
He et al. (2021)	China	205	age 18–70 years, hypertension but not > = 180/120, BMI, 24–40 kg/m2, non-diabetic	CER	103	50.7	43	60	28.7	1000/1200 kcal/day (f/m)	26
				PF	102	50.2	44	58	28.7	500/600 kcal/day (f/m) on 2 fast day, and usual diet on 5 days per week	
Pavlou et al. (2023)	USA	75	aged 18 to 80 years with obesity 30–50 kg/m2 and T2DM	CER	25	55	8	17	38	reduced their energy intake by 25% of their energy needs every day	26
				TRE	25	56	7	18	39	16-hour fasting and 8 h eating	
				Usual	25	54	7	18	39	usual eating	
Lin et al. (2023)	USA	90	age 18–65 years, BMI 30–50 kg/m2, stable weight, no diabetes, non-smoker	CER	30	44	6	24	NA	25% energy restriction daily	52
				TRE	30	44	5	25	NA	16 h fasting and 8-hour eating between noon and 8:00p.m.	
				Usual	30	44	5	25	NA	Eating over period of 10 or more hours per day	
Haganes et al. (2022)	Norway	66	women (36.2 ± 6.2 years with overweight/obesity	TRE	33	36.2	0	33	31.8	energy intake limited to a < 10-hours eating window every day	7
				Usual	33	36.4	0	33	33.1	Usual diet	
Akasheh et al. (2020)	USA	54	age: 18–65 years, BMI 25–40 kg/m^2^, non-diabetic, no history of cardiovascular disease, and nonsmoker	ADF	11	NA	NA	NA	NA	Consume 25% of energy needs on the fast day (500 kcal) and 125% of energy needs on feast days	26
				CER	15	NA	NA	NA	NA	Consume 75% of energy needs ( 1500 kcal) every day	
				Usual	17	NA	NA	NA	NA	Usual diet	
Arciero et al. (2022)	USA	41	aged (30–65 years), healthy nonsmoking men and women, no history or current cardiovascular or metabolic disease, BMI > 27.5 kg/m^2^	CER	19	50.7	33	12	7	1200/1500 kcal/d (f/m)	8
				PF	20	49.7	32.4	14	6	500 kcal/day for the two consecutive fasting days	
Miranda et al. (2018)	USA	42	18–65 years, BMI 25.5–39.9 kg/m2, no cardiovascular disease, no diabetes	ADF	20	44	3	17	33	Consume 25% of energy needs on fast days	24
				Usual	22	43	3	18	34.5	Usual diet	
Obermayer et al. (2023)	Australia	46	18 to 75 years and had diabetes	PF	22	65	10	12	33.5	Fasting 3 days a week, reducing their calories on these days by 75%	12
				Usual	24	61	14	10	35	Usual diet	
Razavi et al. (2021)	Iran	75	80 individuals with MetS, age25-60 years, and BMI 25–40 kg/m2	CER	37	43.1	20	14	31.2	Consumed 75% energy needs each day	17
				mADF	38	41.3	21	14	31.3	75% energy restriction during the 3 fast days	
Sundfor et al. (2018)	Norway	112	men and women aged 21 to 70 years with BMI 30 to 45 kg/m2	CER	58	47.5	28	30	35.3	Reduce their energy intake every day (25%)	26
				PF	54	49.9	28	26	35.1	400/600 kcal/day (f/m) on two non-consecutive days and usual diet 5 days a week	
Cienfuegos et al. (2022)	USA	33	BMI 30.0–49.9 kg/m2; age 18–65 years; sedentary non-smoker, non-diabetic	TRE	19	46	1	18	NA	Eat ad libitum from 1 to 7 p.m. daily, and fast from 7 to 1 p.m. (18-h fast)	10
				Usual	14	45	2	12		Usual diet	
He et al. (2022)	China	113	Individuals met three or more metabolic syndrome- MetS criteria	CER	55	41.3	35	20	29.3	restricts to < 26% of energy intake	13
				TRE	55	43	30	25	29.6	16:8 diet (8 h eating and 16 h fasting each day	
Varady et al. (2013)	USA	32	BMI 20–29.9 kg/m2; age 35–65 years	Usual	15	48	3	12	26	Eat ad libitum every day	12
				mADF	15	47	5	10	26	Consumed 25% of energy needs on the fast day, and ate ad libitum on each alternating feed day	
Oh et al. (2018)	South Korea	23	overweight or obese but healthy adults, 32 to 40 years	Usual	10	40.6	4	6	26.3	Usual diet	8
				mADF	13	32.9	3	10	27.6	Consumed 25% (400–500 kcal) of energy need in 3 days alternately on fast days	
Maroofi et al. (2020)	Iran	88	men and women with a BMI > 25 kg/m2, fasting plasma TG 150–400 mg/dL	CER	44	45.2	15	29	32.4	Consume 70% of the estimated total energy needs	8
				PF	44	44	10	30	31.6	30% of daily calories requirement) for 3 days per week	
Kunduraci et al. (2020)	Turkey	70	metabolic syndrome patients, aged18-65years, BMI) > 27 kg/m2	CER	33	48.76	15	18	32.8	reduction of 25% from habitual energy intake	12
				TRE	32	47.44	16	16	36.58	16 h fasting and 8 h feast	
Guo et al. (2021)	China	46	aged 30 to 50 years, with metabolic syndrome, no CVD, no chronic diseases	PF	21	40.2	10	11	28	a 75% of energy restriction for 2 non consecutive days a week and an ad libitum diet on 5 days	8
				Usual	18	42.7	11	7	27.7	routine diet	
Domaszewski et al. (2022)	Poland	46	Men, age 65-74years old, Nonsmoking, BMI; 25–29.9 kg/m2	TRE	23	69.3	23	0	28	entirely abstaining from food for 16 h a day	6
				Usual	23	69.6	23	0	28.38	Usual diet	
Manoogian et al. (2022)	USA	137	21–65 years old, firefighters, had at least one cardiometabolic risk factor	TRE	70	41.07	60	10	27.77	10-hours TRE	12
				Usual	67	39.6	65	2	27.65	Standard eating	
Fagundes et al. (2023)	Brazil	36	Women age 18 -59y with a body mass index >= 25 kg/m2, no chronic disease(e.g.,diabetes, hypertension, chronicrenal failure)	CER	12	31.1	0	12	30.1	Caloric restriction ranged from 513 to 770 kcal/d	8
				TRE	24	36.2	0	24	30.5	8 -h eating window and 16 h of fasting every day caloric restriction ranged from 513 to 770 kcal/d	
Liu et al. (2023)	China	38	aged 18–22 years, BMI 18.5–23.9 kg/m2, no underlying diseases	TRE	19	20.3	0	19	21.6	Eating for 8 h and fasting for the remainder of the day	8
				Usual	19	20.1	0	19	20.32	maintain their usual lifestyle	
Teong et al. (2023)	Australia	209	aged 35-75years, score > 12 on the Australian Type 2 Diabetes Risk Assessment Tool	CER	83	58	34	49	35	30% reduction of energy requirements daily	26
				PF	85	57	36	49	34.7	Fasting on three non-consecutive days per week, and ad libitum eating on other days	
				Usual	41	59	19	22	33.8	standard care	
Suthutvoravut et al. (2023)	Thailand	46	aged1 8 to 65 years, diagnosed with IFG(i.e.,FPG of 100-125 mg/dL, BMI > = 25 kg/m2	TRE	24	55.5	8	16	29.2	15 h fasting	12
				Usual	22	55.2	6	16	30.3	usual eating	
Lowe et al. (2020)	USA	50	ages 18–64 BMI 30 kg/m2–40 kg/m2, non diabetic	TRE	25	43.3	13	12	31.5	16 h fasting and 8-hours eating	12
				Usual	25	44.4	15	10	31.3	3 structured meal per day	
Carter et al. (2016)	Australia	63	>=18years) withT2DM, BMI > 27 kg/m2)	CER	32	16	16	62	36	1200–1500 kcal/day	12
				PF	31	14	17	62	35	400–600 kcal/day on 2 fast days and regular diet on 5 feed days	
Pinto et al. (2020)	UK	45	non-smoker aged 35–75 years, with a high waist circumference (a high risk of cardiometabolic disease), no diabetes, no cardiovascular disease	CER	22	56	6	16	31.1	daily 500 kcal deficit	4
				PF	21	50	5	15	31.8	consume 600 kcal on 2 consecutive days per week	
Stekovic et al. (2019)	Austria	60	35–65 Years, BMI 22.0–27.0 kg/m2	ADF	28	48	12	17	25.51	eat every second-day ad libitum, but to completely exclude foods on the fast days	4
				Usual	29	50.5	11	17	25.37	usual diet	
Schabel et al. (2018)	Germany	150	women and men, BMI > 25 and < 40 kg/m2, age 35-65y, nonsmokers	CER	49	50.5	31.2	24	25	5:2 diet: consume 80% of the individual energy requirement daily	12
				PF	49	49.4	32	24	25	restrict to 25% on 2 non-consecutive days per week	
				Usual	52	50.7	31.1	27	25	usual diet	
Gray et al. (2021)	Australia	121	females aged > 18y with a previous diagnosis of GDM during pregnancy and a current BMI > 25 kg/m2, no diabetes, or other illness or disease	CER	60	40.2	0	60	32.6	follow a diet of 1500 kcal per day for 7 days a week	52
				PF	61	39.3	0	61	34.8	follow 500 kcal per day for 2 non-consecutive days each week	
Bhutani et al. (2013)	USA	41	age 25–65 years, BMI 30–39.9 kg/m2, non-smoker non-diabetic; no history of cardiovascular disease	ADF	25	42	1	24	35	Consumed 25% of their energy needs on the fast days	12
				Usual	16	49	1	15	35	Regular diet	
Cho et al. (2019)	South Korea	31	Age 20–65 years; BMI) > = 23.0 kg/m2, stable weight, non-diabetic, no chromic disease	Usual	5	42.6	3	2	25.8	Usual diet	8
				mADF	8	33.5	2	5	27.8	Consumed 25% of their daily energy needs (500 kcal) on fast days	
Carter et al. (2019)	Australia	137	aged > 18, with Type 2 diabetes, BMI > 27 kg/m2	CER	67	61	29	38	37	Followed a diet of 5000 to 1200-1500 kcal/day	104
				PF	70	61	31	39	35	followed a diet of 500–600 kcal/ day) for 2 days per week and usual diet for the other 5 days	
Headland et al. (2019)	Australia	222	overweight and obese adults, ages18–72years	CER	104	51.7	19	85	33.4	4200 kJ/ day for women and 5040 kJ/day for men energy restriction	52
				PF	118	47.5	21	97	32.9	(2100 kJ/day for women and 2520 kJ/day for men energy restriction of 2 day /week and usual diet for 5days	
Coutinho et al. (2018)	Norway	35	Adults (18–65 years of age, with obesity (30 < BMI < 40 kg/m2), non-diabetes	CER	14	39.1	2	12	35.1	energy restriction (33% reduction of the estimated energy needs;	12
				PF	14	39.4	4	10	35.6	3 non-consecutive days of partial fasting per week (550 and 660 kcal/day for women and men, respectively)	
Byrne et al. (2018)	Australia	41	males aged 25–54 years, with a body mass index classified as obese (30–45 kg/m2)	CER	23	39.4	23	0	34.3	33% reduction in energy intake	16
				mADF	24	39.8	24	0	34.5		
Harvie et al. (2013)	USA	77	Overweight women aged 20–69 years, BMI 24–45 kg/m2, no diabetes, no CVD	CER	40	47.9	0	37	32.2	a 25% (6000 kJ/d energy restriction for 7d/ week)	12
				PF	37	45.6	0	40	29.6	25% energy restriction for consecutive 2 days and al libitum in for 5 days per week	
Parvaresh et al. (2019)	Iran	70	adults with MeS, aged 25–60 and overweight (BMI 25–40 kg/m2)	CER	34	46.4	20	14	31.6	consumed 75% of their energy need each day	8
				mADF	35	44.6	21	14	31.1	consume a very low-calorie diet (75% energy restriction) during the 3 fast days	
Trepanowski et al. (2018)	USA	79	aged 18-65y, BMI 25–40 kg/m2, nonsmoker, non-diabetes or CVD	CER	29	44	6	23	35	consumed 75% of energy needs everyday	24
				Usual	25	44	4	21	34	Usual diet	
				mADF	25	46	3	22	34	Consuming 25% of energy needs fast day and 125% on eat day	
Lin et al. (2022)	Taiwan	63	women ages 40–65 y, BMI > = 24 kg/m2 or waist circumference > 80 cm	TRE	30	50.1	0	30	25.9	8 h of eating time and fasting for 16 h)	8
				Usual	33	54.2	0	33	25.7	unrestricted eating time)	
Gabel et al. (2019)	USA	43	age 18 to 65 years old, had a BMI of 25.0 to 39.9 kg/m2, insulin-resistant, no type 2 diabetes or cardiovascular disease	CER	17	42	4	13	36	every day 75% intake energy need	52
				Usual	15	41	4	11	35	every day usual diet intake	
				mADF	11	43	2	9	34	Fast day: 125% intake, Fast day: 25% intake energy need	
Che et al. (2021)	China	120	age 18–70 with type 2 diabetes, BMI > = 25Â kg/m2	TRE	60	48.2	31	29	26.42	The 10-h TRF group fed freely from 8:00 to 18:00 and fasted from 18:00 to 8:00 daily (a 14-h fast)	12
				Usual	60	48.8	34	26	26.08	maintain their normal diet	
Chow et al. (2020)	USA	22	overweight or obese (18–65 years, BMI > = 25 kg/m2), non-diabetic	TRE	13	46.5	2	9	33.8	16 h fasting and 8-hour eating window for ad libitum intake	12
				Usual	9	44.2	1	8	34.4	eat ad libitum per their usual habits	
Harvie et al. (2011)	UK	107	premenopausal women aged 30-45years, BMI 24–40 kg/m2, non smoker, no diabetes or other chronic diseases	CER	54	40	0	54	30.5	25% energy restriction for 7 days per week	24
				PF	53	40.1	0	53	30.7	25% energy restriction for 2 day and no restriction for 5 days per week	
Catenacci et al. (2016)	USA	29	Adults with obesity BMI > = 30 kg/m2, age18–55, nonsmoker, 4.5 kg weight change over past 6 months	ADF	13	39.6	3	10	39.5	zero calorie alternate day fasting	8
				CER	12	42.7	3	9	35.8	a 400 kcal/day deficit from estimated energy requirements	
Liu et al. (2022)	China	139	18 to 75 years of age, BMI 28–45 kg/m2, no diabetes, no chronic disease	CER	70	32.2	35	35	31.3	follow a diet of 1500 to 1800 kcal per day and the women to follow a diet of 1200 to 1500 kcal per day	52
				TRE	69	31.6	35	34	31.8	consume the prescribed calories within an 8-hour period (from 8:00 a.m. to 4:00 p.m.) each day	
Conley et al. (2018)	Australia	24	males aged 55–75, BMI > = 30 kg/m2 and stable weight, non-diabetic	CER	12	67.1	12	0	36.2	follow a continuous daily energy-restricted diet (500 calorie daily reduction from average requirement	24
				PF	11	68	11	0	33.4	fasting for two non-consecutive days (restrict calorie intake to 600 calories)	
Domaszewski et al. (2020)	Poland	45	non-smoking women over 60 years of age, average BMI 25 kg/m2	TRE	25	65	0	25	28.99	completely abstaining from food for 16 h a day, from 20:00p.m. to 12:00a.m. (the next day)	6
				Usual	20	66	0	20	26.99	usual diet	
Beaulieu et al. (2020)	UK	46	Women aged between 18 and 55y, BMI between 25.0 and 34.9 kg/m2	ADF	24	35			29.4	on fast days, volunteers consumed 25% of their daily energy requirements	12
				CER	22	34			28.9	participants consumed 75% of their daily energy requirement each day	
Cienfuegos et al. (2020)	USA	58	m/f age 18–65, BMI 30–49.9 kg/m2, sedentary, non-diabetic,	TRE	20	47	19	1	37	eat ad libitum from 1 to 7 pm daily, and fast from 7 to 1 pm (18-hfast)	8
				Usual	19	45	17	2	36	usual diet	
Castela et al. (2022)	Norway	28	adults (20–55 years), with obesity	CER	14	39.1	2	12	35.1	every day 33% reduction of the energy needs)	12
				PF	14	39.4	4	10	35.6	3 non consecutive days of partial fasting per week, (consume 550 / 660 kcal/day (f/m)	
Steger et al. (2021)	USA	35	21-65years, BMI 25–35 kg/m2, weight stable	CER	17	48	3	14	31.4	continuous/daily energy restriction consisted of 1200 to1600kcal	12
				PF	18	43.4	5	13	31.1	IER with 3 days of a very-low energy diet (550 to 800 kcal/d 3 days per week) and 4 days of normal eating	
Mena-Hernandez et al. (2024)	Mexico	28	men and women between 18 and 50 years old; BMI > 25 kg/m2; stable body weight for three months before the study	TRE	9	26	5	12	32	16/8 protocol	4
				Usual	8	26	5	12	32	Usual diet	4
Sukkriang et al. (2024)	Thailand	66	BMI > = 25 kg/m2, age 30–60 years old, with type 2 diabetes mellitus	TRE	33	46	13	20	32	16/8 protocol	12
				Usual	33	44	15	18	32	usual diet	12
Hooshiar et al. (2024)	Iran	49	women aged 18-50years, with a BMI 25–40, and normal menstrual cycles of 21-35days	CER	24	32	0	24	32	daily energy restrictions	8
				mADF	25	32	0	25	32	During fasting days, participants only consuming quarter of their needs	8
Herz et al. (2024)	Germany	18	Healthy aged 18–65 years with a BMI > = 20 kg/m2 and no cardiac problem	ADF	8	25			25	fasting periods occurring on alternate days, the participants abstained from food and beverages for 24 h	8
				TRE	11	26			25	16/8 protocol, fasted for 16 h and remaining 8 h eating	8
Quist et al. (2024)	Denmark	100	age 30–70 years with either overweight (BMI > = 25 and concomitant prediabetes (i.e., glycated haemoglobin) 39–47 mmol/mol) or obesity (i.e., BMI > = 30) [HbA1c with or without prediabetes]	TRE	46	46	18	32	34	10-h per-day eating window	13
				Usual	46	59	16	34	34	usual eating	13

ADF- alternate fasting, CER- Continuous energy restrictions, mADF- modified alternate day fasting, PF- Periodic fasting, TRE- Time restricted eating, BMI- body mass index

### Risk of Bias

**Fig. 2:**
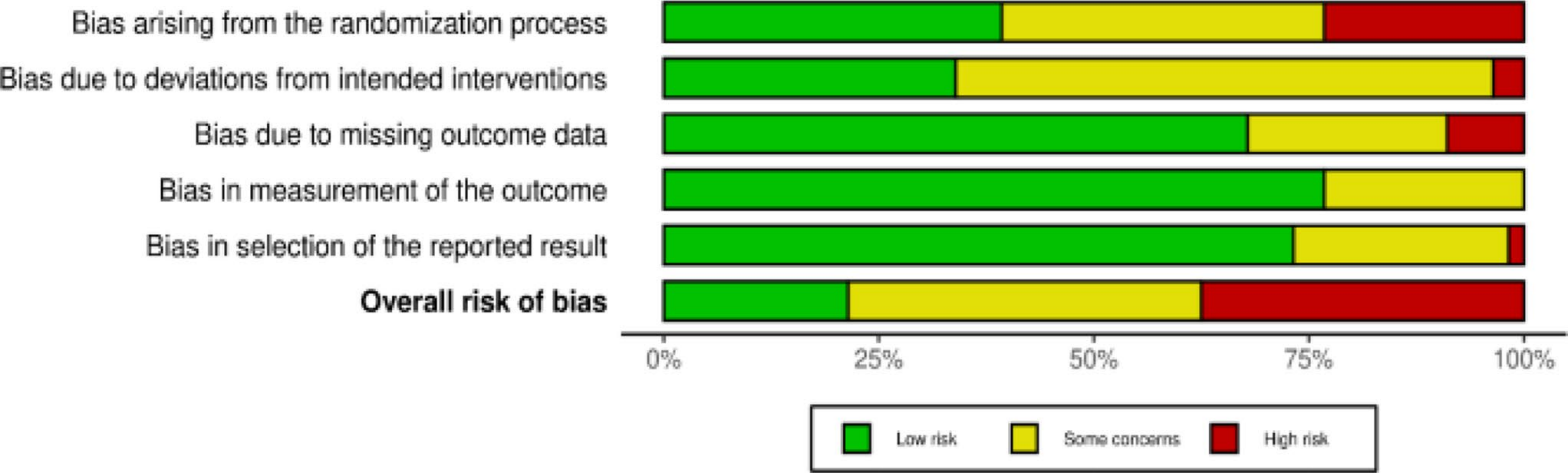
Risk of bias (Summary)

Out of the 56 RCTs, 21 (37.5%) studies were determined to have an overall high risk of bias while 12 (21.4%) studies were rated as overall low risk of bias (Fig. 2). The most common source of bias was related to the randomisation process (high risk, *n* = 13; some concern, *n* = 21) followed by bias due to missing outcome data (high risk, *n* = 5; some concern, *n* = 13). Detailed risk of bias assessment results is presented in Supplementary Fig. S1.

### Certainty of Evidence and Intervention Classifications

**Fig. 3:**
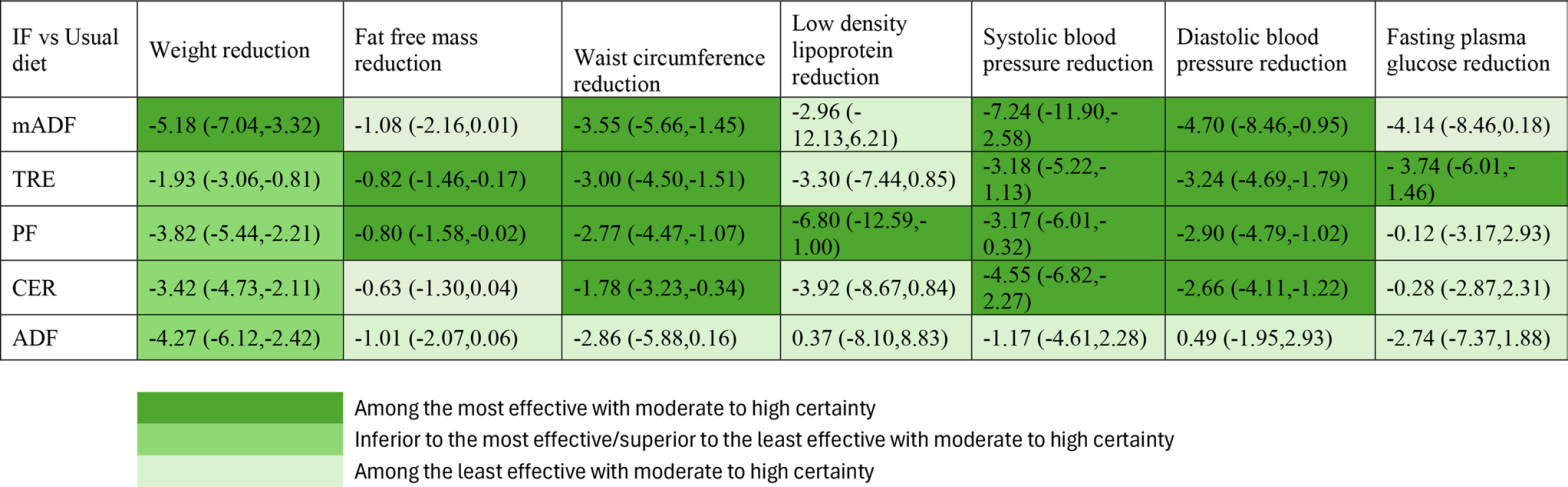
The summary of results network meta-analysis of intermittent fasting regimes (mean difference with 95% CI) in comparison with usual diet for all outcomes along with ranking by new GRADE certainty of evidence framework. Note: mADF = modified alternate day fasting; ADF = alternate day fasting; CER = continuous energy restriction; PF = periodic fasting; TRE time restricted eating

The GRADE assessment details for all outcomes are presented in supplementary Tables S1 A-G. Figure 3 and supplementary Table S2 presents the classification of all interventions for each outcome based on the new GRADE certainty of evidence framework.

### Comparative Effectiveness of Intermittent Fasting

#### Body Composition

##### Body Weight

**Fig. 4:**
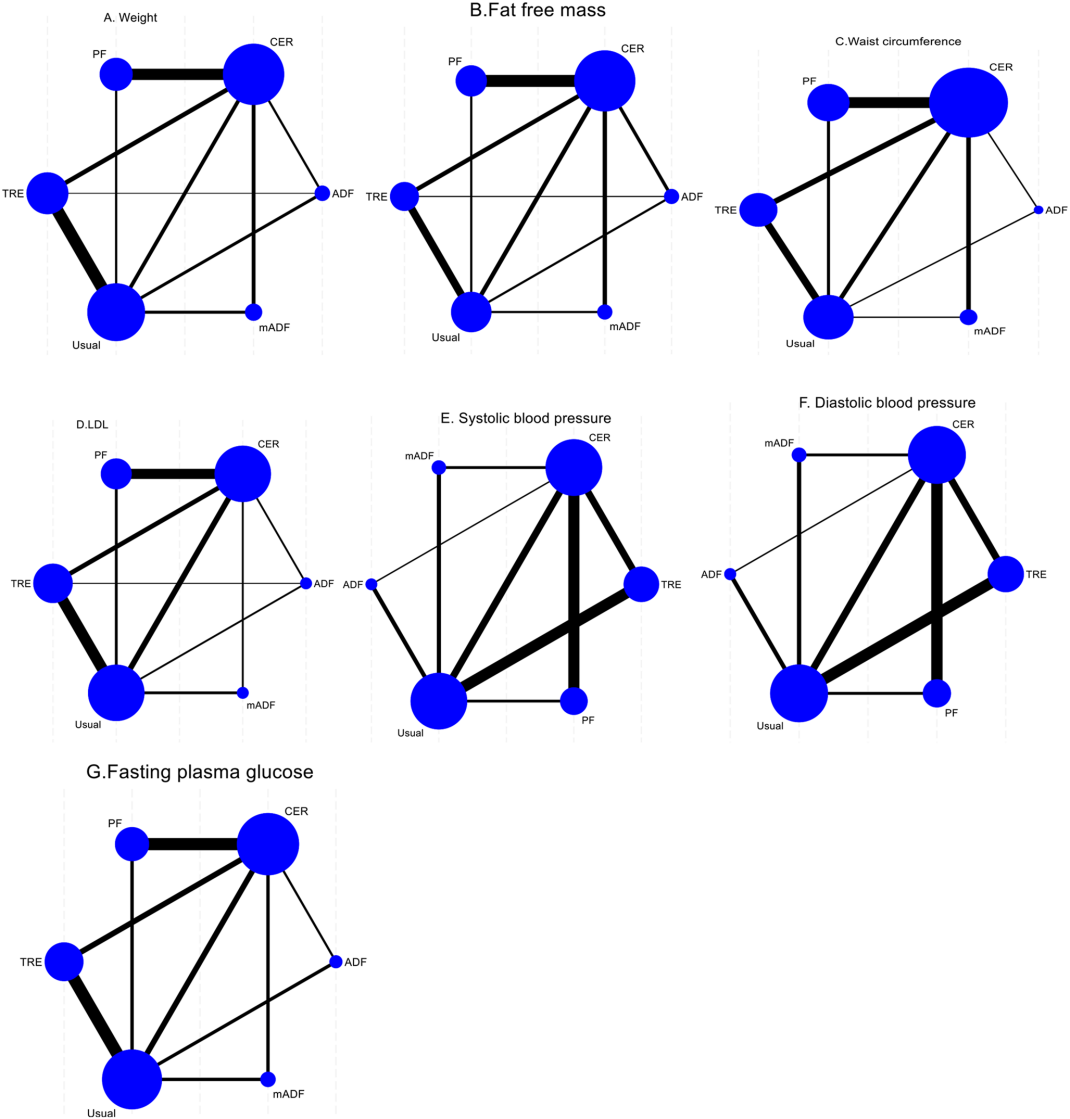
Network plots of the direct comparisons between intermittent fasting interventions from head-to-head trials for the outcomes: (**A**) Weight; (**B**) Fat free mass; (**C**) Waist circumference; (**D**) LDL-cholesterol; (**E**) Systolic blood pressure; (**F**) Diastolic blood pressure; (**G**) Fasting plasma glucose. The sizes of nodes correspond to the number of participants randomized to the intermittent fasting methods and the width of line corresponds to the number of studies. Note: mADF = modified alternate day fasting; ADF = alternate day fasting; CER = continuous energy restriction; PF = periodic fasting; TRE time restricted eating

A total of 52 studies reported weight change after intermittent fasting intervention with a total of 3241 participants. Most of the 52 comparisons were between CER vs. PF (*n* = 14) followed by TRE vs. usual diet (*n* = 14) (Fig. 4A and Supplementary Table S3). The inconsistency analysis revealed the absence of global inconsistency (Supplementary Fig. S2A) and local inconsistency (Supplementary Table S4). Compared to TRE, mADF (MD= -3.24 kg, 95% CI -5.29 to − 1.20, high certainty evidence) effective intervention in reducing weight.

**Fig. 5:**
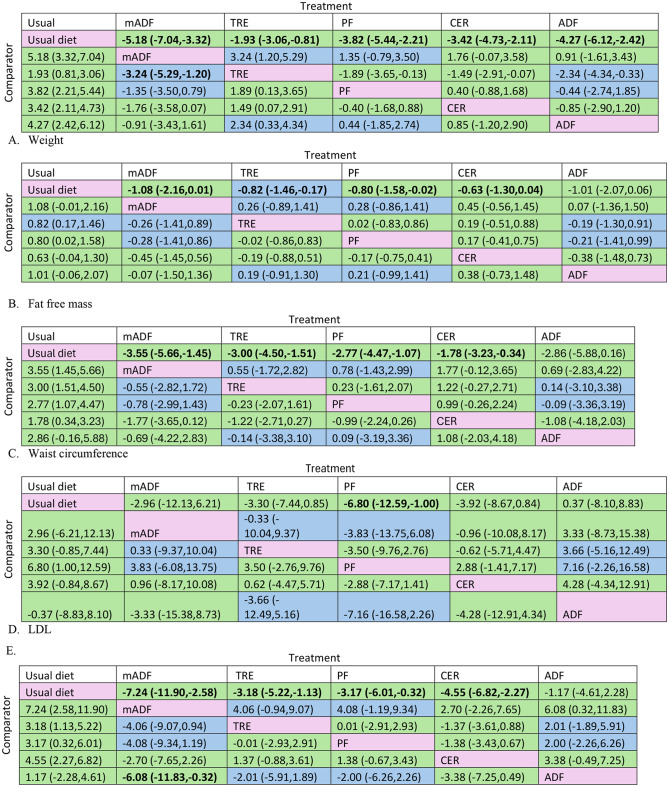

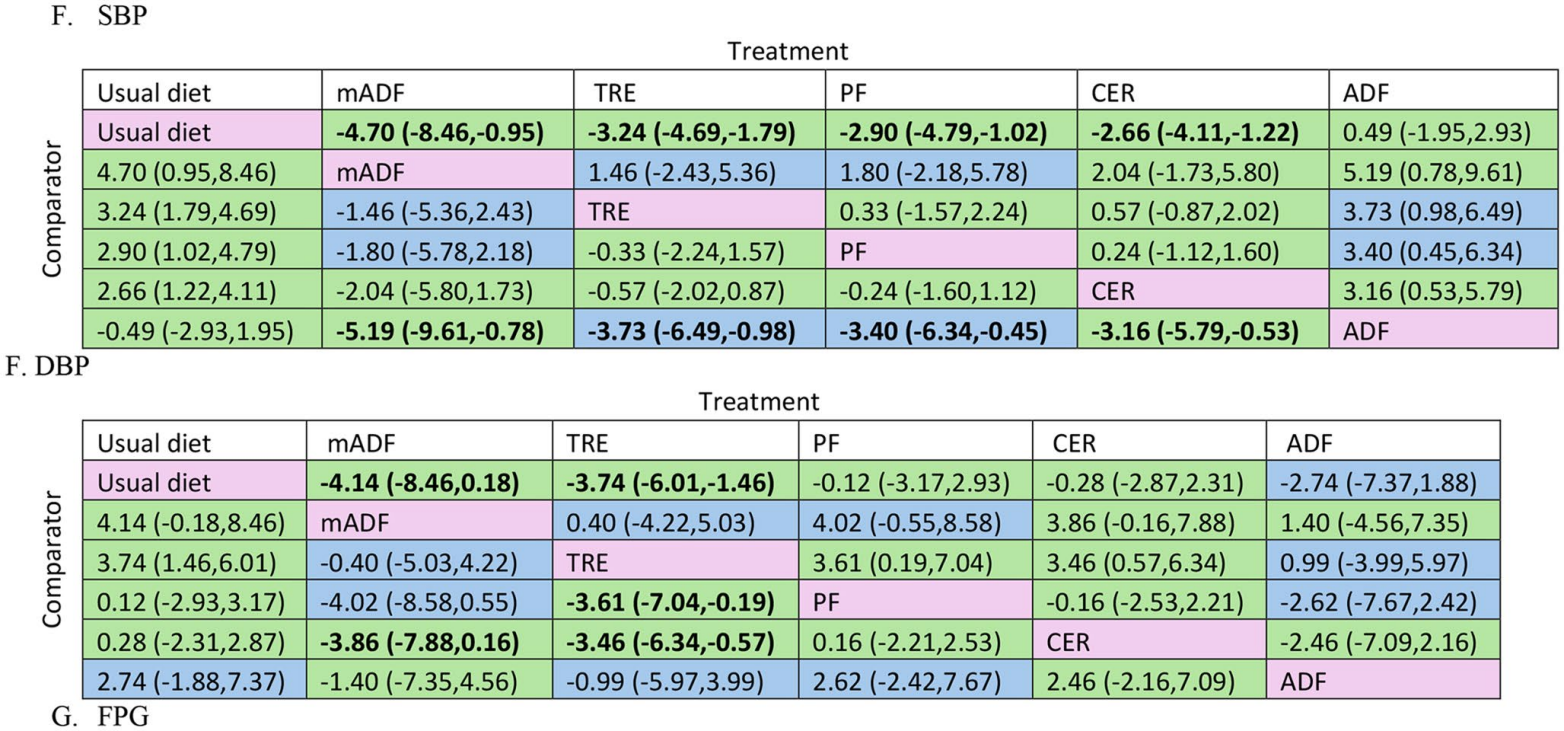
Intermittent fasting network meta-analysis results (mean difference with 95% CI) with corresponding GRADE certainty of evidence for: Weight in kg (**A**); Fat-free mass in kg (**B**); Waist circumference in cm (**C**); Low density lipoprotein-LDL in mg/dL (**D**); Systolic blood pressure -SBP in mmHg (**E**); Diastolic blood pressure - DBP in mmHg (**F**); Fasting plasma glucose– FPG in mg/dL (**G**). Values in bold indicate a statistically significant effect. Colour coding indicates the GRADE certainty of evidence: green = high certainty, blue = moderate certainty. Note: mADF = modified alternate day fasting; ADF = alternate day fasting; CER = continuous energy restriction; PF = periodic fasting; TRE time restricted eating

When compared to usual diet mADF (MD=-5.18 kg; 95% CI: -7.04 to − 3.22, high certainty evidence), ADF (-4.27 kg; -6.12 to -2.42, high certainty evidence), PF (-3.82 kg; -5.44, -2.21, high certainty evidence), CER (-3.42 kg; -4.73 to -2.11, high certainty evidence), and TRE (-1.93 kg; -3.06, -0.81, moderate certainty evidence) significantly reduced body weight (Fig. 5A, Supplementary Table S1).

Among the intermittent fasting methods with high or moderate certainty of evidence, compared to a usual diet, mADF was the most effective, whereas CER, TRE, ADF and PF were among the interventions with intermediate effectiveness in reducing body weight compared to usual diet (Fig. 3 and Supplementary Table S2, Supplementary Fig. S3A).

##### Fat Free Mass

Change in fat-free mass was reported in 32 studies with a total of 2045 participants. Most comparation were between PF vs. CER (*n* = 10), followed by TRE vs. usual diet (*n* = 6) (Fig. 4 and Supplementary Table S3). Both the global inconsistency test (Supplementary Fig. S2B) and the local inconsistency test supported the consistency of the direct and indirect estimates (Supplementary Table S4).

Compared to usual diet, TRE (MD= -0.82 kg; 95% CI: -1.46 to -0.17, moderate certainty evidence), PF (-0.80 kg; -1.58 to -0.02, high certainty of evidence) significantly reducing fat-free mass (Fig. 5B and Supplementary Table S1). Among intermittent fasting methods with high or moderate certainty of evidence, compared to a usual diet, TRE, and PF were the most effective for fat free mass reduction, whereas mADF and ADF was not better than usual diet (Fig. 3, Supplementary Table S2, Supplementary Fig. 3B).

##### Waist Circumference

Most of the 22 comparisons were between CER vs. PF (*n* = 7), CER VS mADF(*n* = 3) and TRE vs. usual diet (*n* = 3) (Fig. 4C and Supplementary Table S3). The global and local inconsistency test indicated no violation of the consistency assumption for direct and indirect estimates (Supplementary Fig. 2C and Supplementary Table S3).

Compared to usual diet with high certainty of evidence, mADF (MD= -3.55 cm; 95% CI: -5.66 to -1.45), CER (-1.78 cm; -3.23, -0.34), PF (-2.77 cm; -4.47, -1.07) and TRE (-3.00 cm; -4.50, -1.51) significantly reduced waist circumference (Fig. 5C and Supplementary Table S1). However, there were no statistically significant differences among the other comparisons (Fig. 5C). Among the intermittent fasting methods with high or moderate certainty of evidence, compared to a usual diet, mADF, CER, TRE, and PF were the most effective for fat free mass reduction, whereas ADF was probably among least effective (not better than usual diet) (Fig. 3, Supplementary Table S2, Supplementary Fig. S3C).

#### LDL Cholesterol

Change in LDL cholesterol levels were reported in 35 articles with a total of 2488 participants, and most comparisons were TRE vs. usual diet (*n* = 10) and CER vs. usual diet (*n* = 9) (Fig. 4D and Supplementary Table S3). With high certainty of the evidence, PF (MD= -6.80 mg/dL; 95% CI: -12.59, -1.00) was associated with a significant reduction in LDL level compared to usual diet; however, there were no significant differences among the other comparisons (Fig. 5D and Supplementary Table S1). Among the intermittent fasting methods with high or moderate certainty of evidence, compared to a usual diet, PF was among the most effective, while mADF, CER, TRE and ADF were not better than usual diet for LDL reduction (Fig. 3 and Supplementary Table S2, Supplementary Fig. S3D).

#### Blood Pressure

##### Systolic Blood Pressure (SBP)

SBP was reported in 27 studies, with a total of 1852 participants. Most of the 27 comparisons were CER vs. usual diet (*n* = 7) and TRE vs. usual diet (*n* = 6). With high certainty, mADF (-6.08 mmHg; -11.83 to -0.32) was more effective in reducing SBP compared to ADF. Compared to usual diet with high certainty of evidence, mADF (MD= -7.24 mmHg; 95%CI: -11.90 to -2.58), CER (-4.55 mmHg; -6.82 to -2.27), PF (-3.17 mmHg; -6.01 to -0.32) and TRE (-3.18 mmHg; -5.22 to -1.13) significantly reduced SBP (Fig. 5E and Supplementary Table S1). Among the intermittent fasting methods with high or moderate certainty of evidence, compared to a usual diet, mADF, CER, TRE, and PF were the most effective for SBP reduction, whereas ADF was not better than usual diet (Fig. 3, Supplementary Table S2, Supplementary Fig. S3E).

##### Diastolic Blood Pressure (DBP)

DBP was reported in 27 studies, with a total of 1861 participants, and most compared CER vs. usual diet (*n* = 7) and TRE vs. usual (*n* = 6). Compared to ADF, mADF (-5.19 mmHg; -9.61 to -0.78, high certainty evidence), TRE (-3.73 mmHg; -6.49 to -0.98, high certainty evidence), PF (-3.40 mmHg; -6.34 to -0.45, high certainty evidence) are more effective in reducing DBP. Compared to usual diet with high certainty of evidence, mADF (MD= -4.70 mmHg; 95%CI: -8.46 to -0.95), CER (2.66 mmHg; -4.11 to -1.22), PF (-2.90 mmHg; -4.79 to -1.02) and TRE (-3.24 mmHg; -4.69 to -1.79) significantly reduced DBP (Fig. 4F and Supplementary Table S1). Among the intermittent fasting methods with high or moderate certainty of evidence, compared to a usual diet, mADF, CER, TRE, and PF were the most effective for DBP reduction (Fig. 3, Supplementary Table S2, Supplementary Fig. S3F).

#### Fasting Plasma Glucose (FPG)

A total of 36 studies reported FPG change after intermittent fasting intervention involving a total of 2428 participants. Most comparison were TRE vs. usual diet (10) and PF vs. CER (*n* = 9) (Fig. 4 and Supplementary Table S3). The inconsistency examination revealed the absence of global inconsistency and local inconsistency (Supplementary Fig. S2G and Supplementary Table S4). With high certainty, TRE (-3.46 mg/dL; -6.34, -0.57) are more effective than CER in reducing FPG. Similarly, TRE (-3.61 mg/dL; -7.04, -0.19) with high certainty is effective in reducing FPG compared to PF. Relative to usual diet with high certainty of evidence, TRE (-3.74 mg/dL; -6.01, -1.46) significantly reduced FPG (Fig. 5G and Supplementary Table S1). Among the intermittent fasting methods with high or moderate certainty of evidence, compared to a usual diet, TRE was probably the most effective; mADF, PF, and ADF probably among least effective intermittent fasting methods (not better than usual diet) for FPG reduction (Fig. 3 and Supplementary Table S2, Supplementary Fig. S3G).

### Sensitivity Analysis

#### Excluding Studies with Participants with Diabetes

Compared to the main analysis, the effects of intermittent fasting on body weight, FPG, SBP, and DBP remained similar in magnitude and direction. However, the previously significant effects of mADF and CER on waist circumference was no longer observed. Additionally, the positive effects of PF on waist circumference and fat-free mass were no longer statistically significant (Supplementary Fig. S4).

#### Excluding Studies with High-Risk of Bias

The size and direction of the network estimates for weight, FPG and SBP were consistent with the full analysis in this sensitivity analysis. However, the previously significant effects of PF on waist circumference and LDL, and the effect of mADF on DBP and TRE on fat free mass were no longer significant. Conversely, the effect of CER on fat free mass was statistically significant among this sub-set of higher quality studies (Supplementary Fig. S5).

#### Excluding Studies with Short Intervention Durations

The size and direction of the network estimates for weight, waist circumference and LDL cholesterol were in line with the full analysis. But the effects of mADF on SBP and DBP, and the effect of TRE on FPG and fat free mass were no longer significant. Conversely, the effect of CER on fat free mass and the effect of mADF on FPG were statistically significant (Supplementary Fig. S6).

## Discussion

This systematic review and network meta-analysis synthesised the evidence on the effect of various intermittent fasting methods on cardiovascular disease risk factors using 56 randomised controlled trials conducted between 2013 and 2024. The findings indicated that different intermittent fasting modalities, when compared to a usual diet, significantly reduced body weight, fat-free mass, waist circumference, LDL levels, blood pressure, and FPG. The mADF was found to be the most effective intervention, with high or moderate certainty of the evidence, for the reduction of cardiovascular risk factors including SBP, DBP, weight, and waist circumference. Compared to a usual diet, time-restricted eating was the most effective intermittent fasting regimen for the reduction of fat-free mass and FPG. Moreover, PF was superior to a usual diet in reducing LDL levels. ADF did not show convincing evidence of superiority to a usual diet to reduce cardiovascular risks except for weight. When comparing each other, mADF is more effective than ADF in reducing SBP and DBP. Similarly, TRE and PF are more effective than ADF in reducing DBP. Additionally, TRE is more effective in reducing FPG compared to PF and CER.

The results of this network meta-analysis revealed a significant reduction in body weight across intermittent fasting methods compared to the usual diet, with ADF, mADF, PF, and TRE demonstrating notable effects compared to a usual diet. Likewise, compared to the usual diet, three intermittent fasting methods - mADF, PF, and TRE - significantly reduced waist circumference, a crucial marker of central adiposity. These results align with previous research [11–14] highlighting the weight management potential of intermittent fasting method. These findings reinforce the potential of intermittent fasting as a viable intervention for weight or waist circumference reduction.

One of the concerns surrounding intermittent fasting is its potential undesirable effect on fat-free mass loss which can impair physical function and cardiometabolic health [15, 89]. However, the evidence regarding this effect was not conclusive. Some studies reported no impact on fat free mass [13, 16], while others indicated an increase in fat-free mass [17], and yet other showed intermittent fasting significantly reduced fat-free mass [15]. Our study revealed a significant reduction in fat-free mass in two intermittent fasting methods (TRE and PF), but no significant reduction in other two intermittent fasting methods (mADF, and ADF). But compared to CER, there is no significant difference in fat-free mass reduction in most intermittent fasting methods. It is important to note that reductions in fat free mass are common across various weight loss strategies [90]. This underscores the necessity for a nuanced understanding of the physiological changes associated with different intermittent fasting strategies.

LDL-cholesterol, as a component of lipid profiles, is another important cardiovascular disease risk factor. Our study found variations in effects on LDL-cholesterol among the different intermittent fasting method. Notably, the PF regimen showed a significant reduction in LDL levels. This aligns with a previous study [18]. However, other studies have not found a consistent effect of intermittent fasting on LDL reduction compared to a usual diet [11, 12, 14].

Our study found significant reductions in both SBP and DBP across multiple intermittent fasting methods, including mADF, PF, and TRE. These findings are partially consistent with previous meta-analyses. Some reported a significant decrease in DBP with intermittent fasting [11, 12], while others did not [13]. Similarly, one meta-analysis found a decrease in DBP with intermittent fasting [11], whereas others showed no effect [13, 14]. These variations highlight the need for further research and potentially personalised approaches to intermittent fasting, considering individual health conditions and risk factors. Another potential benefits of intermittent fasting could be for glycemic control (reduction of blood glucose level). Our study found that TRE method significantly reduced FPG levels. However, these findings are not entirely consistent with previous research. While some meta-analyses reported significant FPG reductions with intermittent fasting [11, 14], others did not observe a significant difference compared to usual eating [13]. The discrepancy could potentially be explained by differences in the duration of the intervention (with some having shorter duration studies) [12–14] and number of studies (with some having fewer studies) [12–14], as well as some analyse lumped different intermittent fasting method together [11, 12].

The underlying mechanisms of the effect of fasting on cardiovascular risk factors are thought to be mediated, at least in part, by the metabolic switch from carbohydrate utilization to fat and ketones oxidation that happens during fasting [9]. Intermittent fasting causes organs to switch between storing and using energy sources [9]. In conventional eating, carbohydrates and fats get stored in the liver, muscles, and fat tissue. But during fasting, the body burns stored glycogen and fat for energy, resulting in more frequent cycling between storing and burning nutrients compared to constant eating and creates metabolic adaptability and weight reduction [91, 92]. This helps the body become more flexible in using energy, leading to various health benefits, including better insulin sensitivity, increased fat burning, and weight loss [93]. However, more research is needed to understand exactly how specific intermittent fasting patterns affect fat breakdown and turnover and how they influence overall calorie burning.

### Strengths and Limitations

This comprehensive systematic review and network meta-analysis employed stringent inclusion and exclusion criteria and included only RCTs. A strength of this review is the ability to compare the relative effectiveness of five commonly used intermittent fasting modalities on a range of cardiovascular disease risk factors, and the certainty of evidence was assessed using the revised version of Cochrane risk of bias assessment tool. This provides valid evidence for decision making and the development of guidance on intermittent fasting. This study incorporated both short-term and long-term studies, and sensitivity analysis was done to assess the robustness of the results. Moreover, in this study, the evidence of certainty has been assessed using the newly validated GRADE framework, which helped to grade the intermittent fasting modalities in a more stringent manner based on a combination of criteria, including effect size, certainty of evidence and SUCRA rankings. Our use of randomized trials strengthens the study’s internal validity but may limit generalizability to real-world settings.

It is essential to note that the lack of direct comparisons between specific intermittent fasting modalities, such as ADF, mADF, TRE, and PF, in our study points towards a gap in the existing literature. The observed risk of bias in 37% of the studies included in our analysis is consistent with the challenges faced by many meta-analyses where the quality of individual studies varies, even though the result remains consistent in the sensitivity analysis. Similarly, the short duration of the included studies might limit the findings, even though the results remain consistent in the sensitivity analysis, except for the effects of mADF on SBP and DBP and the effect of TRE on fat-free mass and FPG, which were no longer significant when excluding studies with short intervention durations. This underscores the importance of interpreting the findings with caution and emphasizes the need for further studies. Future studies should aim to directly compare different intermittent fasting modalities, consider longer-term outcomes, and adhere to rigorous methodologies, including randomization and blinding, to enhance the reliability of results.

## Conclusions

This network meta-analysis compared various intermittent fasting methods and found that mADF and TRE were associated with greater reductions in SBP and DBP compared to ADF, and TRE showed greater effects on FPG compared to PF and CER. PF was more effective than usual diets in lowering LDL cholesterol. Both mADF and ADF were more effective than usual diets in reducing body weight, while TRE was associated with reductions in waist circumference, DBP, FPG, and fat-free mass. Among the methods assessed, mADF showed relatively greater effects across several cardiovascular risk factors. These findings suggest that certain intermittent fasting approaches may hold promise as part of lifestyle strategies to improve cardiovascular risk profiles. However, the results should be interpreted with caution due to high risk of bias as per reviewer, and other limitations such as short intervention duration in many studies. Further high-quality, long-term randomized controlled trials are needed to establish the sustained efficacy and safety of different intermittent fasting methods.

## Key References

- D. Herz, S. Karl, J. Weiß, P. Zimmermann, S. Haupt, R. T. Zimmer, J. Schierbauer, N. B. Wachsmuth, K. Khoramipour, M. P. Erlmann, T. Niedrist, T. Voit, S. Rilstone, H. Sourij, and O. Moser. “Effects of different types of intermittent fasting interventions on metabolic health in healthy individuals (EDIF): A randomised trial with a controlled-run in phase”, Nutrients. 2024;16(8). 10.3390/nu16081114.
- This randomised controlled trial investigated the effect of different intermittent fasting on body composition and metabolic and haematological markers in healthy participants. The data suggest that some fasting interventions might be promising for metabolic health. This reference is ‘of importance’.
- Obermayer, N. J. Tripolt, P. N. Pferschy, H. Kojzar, F. Aziz, A. Muller, M. Schauer, (A) Oulhaj, F. Aberer, C. Sourij, H. Habisch, T. Madl, T. Pieber, (B) Obermayer-Pietsch, V. Stadlbauer, H. Sour. “Efficacy and Safety of Intermittent Fasting in People With Insulin-Treated Type 2 Diabetes (INTERFAST-2)-A Randomized Controlled Trial”, Diabetes Care 2023;46:463–468. 10.2337/dc22-1622.
- This randomised controlled study elucidates the safety and effectiveness of intermittent fasting in type 2 diabetes. Findings show that intermittent fasting has the potential to become a promising therapy option in people with insulin-treated type 2 diabetes. This reference is of ‘outstanding importance’.
- S. Lin, S. Cienfuegos, M. Ezpeleta, K. Gabel, V. Pavlou, A. Mulas, K. Chakos, M. McStay J. Wu, L. Tussing-Humphreys. “Time-Restricted Eating Without Calorie Counting for Weight Loss in a Racially Diverse Population”, Ann Intern Med. 2023; 176(7): 885–895. https://doi.org/10.7326/M23-0052.
- This randomised controlled trial assessed whether time-restricted eating is more effective for weight control and cardiometabolic risk reduction than calorie restriction or control. Time-restricted eating is more effective in producing weight loss when compared with control but not more effective than calorie restriction in a racially diverse population. This reference is ‘of importance’.

## Electronic Supplementary Material

Below is the link to the electronic supplementary material.

Supplementary Material 1

Supplementary Material 2

Supplementary Material 3

Supplementary Material 4

## Data Availability

No datasets were generated or analysed during the current study.
