# Supplementary material for: Intermittent Fasting for the Prevention of Cardiovascular Disease Risks: Systematic Review and Network Meta-Analysis
Source: Curr Nutr Rep. 2025 Jul 24;14(1):93. doi: 10.1007/s13668-025-00684-7 (PMC12289860; doi:10.1007/s13668-025-00684-7)
Supplement: Supplementary file 2 — Supplementary Material 2 [file 13668_2025_684_MOESM2_ESM.docx]

**Supplementary material S2**

**Title: Intermittent fasting for the prevention of cardiovascular disease risks: systematic review and network meta-analysis**

**Key words:** “Intermittent fasting” or “alternate day fasting” or “periodic fasting” or “time restricted eating” or “time restricted feeding” AND “Body weight” or “waist circumference” or “fat-free mass” or “systolic blood pressure” or “diastolic blood pressure” or LDL-c or “fasting blood glucose” AND adults (>18 years old) AND randomised controlled trials

**Databases: Medline, Embase, Cochrane library, Embase, Global Health**

**Ovid MEDLINE(R) and In-Process, In-Data-Review & Other Non-Indexed Citations**
Search Strategy:

| **#** | **Searches** |
| --- | --- |
| 1 | Intermittent Fasting/ |
| 2 | ("alternate day fasting" or whole day fast*).tw. |
| 3 | "alternate day modified fasting".tw. |
| 4 | "periodic fasting".tw. |
| 5 | "periodic eating".tw. |
| 6 | "time restricted eating".tw. |
| 7 | "time restricted feeding".tw. |
| 8 | "time-restricted diet".tw. |
| 9 | "time-restricted fasting".tw. |
| 10 | ("intermittent energy restriction" or "Energy-Restricted Diet").tw. |
| 11 | "intermittent calorie restriction".tw. or Caloric Restriction/ |
| 12 | Blood Pressure/ or Hypertension/ or "systolic blood pressure".tw. |
| 13 | Body Weight/ |
| 14 | Waist Circumference/ or Obesity, Abdominal/ |
| 15 | "fat-free mass".tw. |
| 16 | Hypertension/ or "systolic Blood Pressure".tw. or Blood Pressure/ |
| 17 | "diastolic Blood Pressure".tw. |
| 18 | Body Mass Index/ |
| 19 | Diabetes Mellitus, Type 2/ or "fasting blood glucose".tw. or Blood Glucose/ |
| 20 | "low-density lipoprotein".tw. or Lipoproteins, LDL/ |
| 21 | Metabolic Syndrome/ or Cardiometabolic Risk Factors/ |
| 22 | 1 or 2 or 3 or 4 or 5 or 6 or 7 or 8 or 9 or 10 or 11 |
| 23 | 12 or 13 or 14 or 15 or 16 or 17 or 18 or 19 or 20 or 21 |
| 24 | 22 and 23 |
| 25 | limit 24 to (english language and humans and "all adult (19 plus years)") |

|  |  |  |
| --- | --- | --- |

| **Search Name: Cochrane central** | |
| --- | --- |
| ID | Search |
| #1 | MeSH descriptor: [Intermittent Fasting] explode all trees |
| #2 | (alternate day fasting):ti,ab,kw (Word variations have been searched) |
| #3 | (whole day fasting):ti,ab,kw (Word variations have been searched) |
| #4 | (alternate day modified fasting):ti,ab,kw (Word variations have been searched) |
| #5 | ("periodic fasting"):ti,ab,kw (Word variations have been searched) |
| #6 | ("periodic eating"):ti,ab,kw (Word variations have been searched) |
| #7 | (time restricted eating or time-restricted diet or time-restricted fasting):ti,ab, kw 1593 |
| #8 | ("intermittent energy restriction" or "energy restricted diet" or "energy restricted eating" or "intermittent calorie restriction"):ti,ab,kw |
| #9 | #1 OR #2 OR #3 OR #4 OR #5 OR #6 OR #7 OR #8 |
| #10 | MeSH descriptor: [Waist Circumference] 1 tree(s) exploded |
| #11 | ("systolic blood pressure" or "diastolic blood pressure"):ti,ab, kw |
| #12 | (Cholesterol, LDL):ti,ab,kw (Word variations have been searched) |
| #13 | ("low density lipoprotein cholesterol"):ti,ab,kw |
| #14 | ("Body Weight"):ti,ab,kw |
| #15 | (fat-free mass or abdominal obesity):ti,ab,kw |
| #16 | MeSH descriptor: [Body Mass Index] |
| #17 | MeSH descriptor: [Body Weight] this term only |
| #18 | MeSH descriptor: [Diabetes Mellitus, Type 2] this term only |
| #19 | MeSH descriptor: [Blood Glucose] explode all trees |
| #20 | ("fasting blood glucose level"):ti,ab,kw (Word variations have been searched) |
| #21 | MeSH descriptor: [Metabolic Syndrome] 1 tree(s) exploded |
| #22 | MeSH descriptor: [Cardiometabolic Risk Factors] this term only |
| #23 | ("Cardiovascular Risk Factors"):ti,ab,kw (Word variations have been searched) |
| #24 | #10 OR #11 OR #12 OR #13 OR #14 OR #15 OR #16 OR #17 OR #18 OR #19 OR #20 OR #21 OR #22 OR #23 |
| #25 | #9 AND #24 in Trials |

| **Embase** |  |
| --- | --- |
|  |  |
| ....................................................... | |
| No. | Query |
| #28. | #14 AND #26 AND ([adult]/lim OR [young adult]/lim |
|  | OR [middle aged]/lim OR [aged]/lim OR [very |
|  | elderly]/lim) AND [humans]/lim AND [english]/lim |
| #27. | #14 AND #26 |
| #26. | #15 OR #16 OR #17 OR #18 OR #19 OR #20 OR #21 OR |
|  | #22 OR #23 OR #24 OR #25 |
| #25. | 'cardiometabolic risk factor'/exp/mj OR |
|  | 'cardiovascular risk factor'/exp/mj |
| #24. | 'metabolic syndrome x'/exp/mj |
| #23. | 'non insulin dependent diabetes mellitus'/exp/mj |
| #22. | 'glucose blood level'/exp/mj |
| #21. | 'abdominal obesity'/exp |
| #20. | 'fat free mass'/exp/mj |
| #19. | 'body weight'/exp |
| #18. | 'low density lipoprotein cholesterol'/exp |
| #17. | 'diastolic blood pressure'/exp |
| #16. | 'systolic blood pressure'/exp |
| #15. | 'waist circumference'/exp/mj |
| #14. | #1 OR #2 OR #3 OR #4 OR #5 OR #6 OR #7 OR #8 OR |
|  | #9 OR #10 OR #11 OR #12 OR #13 |
| #13. | 'intermittent fasting'/exp |
| #12. | 'intermittent energy restriction'/exp OR |
|  | 'intermittent energy restriction' OR |
|  | (intermittent AND ('energy'/exp OR energy) AND |
|  | ('restriction'/exp OR restriction)) |
| #11. | 'intermittent calorie restriction'/exp OR |
|  | 'intermittent calorie restriction' OR |
|  | (intermittent AND ('calorie'/exp OR calorie) AND |
|  | ('restriction'/exp OR restriction)) |
| #10. | 'energy-restricted diet' OR ('energy restricted' |
|  | AND ('diet'/exp OR diet)) |
| #9. | 'intermittent energy restriction'/exp OR |
|  | 'intermittent energy restriction' OR |
|  | (intermittent AND ('energy'/exp OR energy) AND |
|  | ('restriction'/exp OR restriction)) |
| #8. | 'time-restricted diet' OR ('time restricted' AND |
|  | ('diet'/exp OR diet)) |
| #7. | 'time restricted feeding'/exp OR 'time restricted |
|  | feeding' OR (('time'/exp OR time) AND restricted |
|  | AND ('feeding'/exp OR feeding)) |
| #6. | 'time restricted eating'/exp OR 'time restricted |
|  | eating' OR (('time'/exp OR time) AND restricted |
|  | AND ('eating'/exp OR eating)) |
| #5. | 'alternate day modified' OR (alternate AND |
|  | ('day'/exp OR day) AND modified AND fast*) |
| #4. | 'whole day' OR (whole AND ('day'/exp OR day) AND |
|  | fast*.tw.) |
| #3. | 'time restricted' OR (('time'/exp/mj OR time) AND |
|  | restricted AND fast*.tw.) |
| #2. | 'alternate day fasting'/exp OR 'alternate day |
|  | fasting' OR (alternate AND ('day'/exp OR day) AND |
|  | ('fasting'/exp OR fasting)) |
| #1. | 'time restricted' OR (('time'/exp/mj OR time) AND |
|  | restricted AND feed*.tw.) |

.......................................................

**Database- Global Health**

| **#** | **Query** |
| --- | --- |
| S17 | (S6 OR S7 OR S8 OR S9 OR S10 OR S11 OR S12 OR S13 OR S14) AND (S5 AND S15) Narrow by Language: - english |
| S16 | (S6 OR S7 OR S8 OR S9 OR S10 OR S11 OR S12 OR S13 OR S14) AND(S5 AND S15) |
| S15 | S6 OR S7 OR S8 OR S9 OR S10 OR S11 OR S12 OR S13 OR S14 |
| S14 | TI ( "Metabolic Syndrome" or "cardiometabolic risk factors" or "cardiovascular riskfacrors" ) OR AB ( "Metabolic Syndrome" or "cardiometabolic risk factors" or"cardiovascular risk facrors" ) |
| S13 | TI ( diabets or type 2 diabetes mellitus or |
|  | "fasting blood glucose " or "blood glucose" ) OR AB ( diabets or type 2 diabetes mellitus or |
|  | "fasting blood glucose" or "blood glucose" ) |
| S12 | TI ( "fat-free mass" OR abdominal obesity ) OR |
|  | AB ( "fat-free mass" OR abdominal obesity ) |
| S11 | TI ( "body mass index" OR "body weight" ) OR AB ( "body mass index" OR "body weight" ) |
| S10 | TI ( low-density lipoprotein cholestrol or cholestrol ) OR AB ( low- |
|  | density lipoprotein cholestrol or cholestrol ) |
| S9 | TI ( "low-density lipoprotein cholestrol" or cholestrol ) OR AB ( "low- density lipoprotein cholestrol" or cholestrol ) |
| S8 | TI ( "systolic bloodpressure" OR "diastolic blood pressure" ) OR AB ( "systolic blood pressure" OR "diastolic blood pressure" ) |
| S7 | DE "blood pressure" OR DE "hypertension" |
| S6 | TI ( "waist circumference" or "waist measurement" ) |
|  | OR AB ( "waist circumference" or "waist measurement" ) |
| S5 | S1 OR S2 OR S3 OR S4 |
| S4 | TI ( "intermittent energy restriction" OR "Energy- Restricted Diet" OR"intermittent calorie |
|  | restriction" ) OR AB ( "intermittent energy restriction" OR "Energy- Restricted Diet" OR"intermittent calorie restriction" ) |
| S3 | TI ( "periodic fasting" OR "periodic eating" OR "time restricted eating" OR "time restricted |
|  | feeding" OR "time- restricted diet" OR "time- restricted fasting" ) OR AB ( "periodic fasting"OR "periodic eating" OR "time restricted eating" OR "time restricted feeding" OR "time-restricted diet" OR "time- restricted fasting" ) |
| S2 | TI ( "intermittent fasting" OR "intermittent fasting" ) |
|  | OR AB ( "intermittent fasting" OR "intermittent fasting" ) |
| S1 | TI ( "alternate dayfasting" or "whole day fasting" or "alternate day modified fasting" ) OR AB ( "alternate day fasting"or "whole day fasting" or "alternate day modified fasting" ) |
