# Supplementary material for: Intermittent Fasting for the Prevention of Cardiovascular Disease Risks: Systematic Review and Network Meta-Analysis
Source: Curr Nutr Rep. 2025 Jul 24;14(1):93. doi: 10.1007/s13668-025-00684-7 (PMC12289860; doi:10.1007/s13668-025-00684-7)
Supplement: Supplementary file 3 — Supplementary Material 3 [file 13668_2025_684_MOESM3_ESM.docx]

**Intermittent fasting for the prevention of cardiovascular disease risks: systematic review and network meta-analysis**

**Supplementary Tables**

[Supplementary Table 1A-G: Grade assessment of certainty of evidence 1](#_Toc185269018)

[Supplementary Table 2: Classification of intermittent fasting interventions 8](#_Toc185269019)

[Supplementary Table 3: The number of studies each treatment comparison for each outcome 13](#_Toc185269020)

[Supplementary Table 4: Local inconsistency assessment 17](#_Toc185269021)

Supplementary Table 1A-G: Grade assessment of certainty of evidence

1. Weight

| Comparison | Within-study bias | Reporting bias | Indirectness | Imprecision | Heterogeneity | Incoherence | Confidence rating |
| --- | --- | --- | --- | --- | --- | --- | --- |
| ADF vs CER | Some concerns | Some concerns | Some concerns | No concerns | No concerns | No concerns | High |
| ADF vs TRE | Some concerns | Some concerns | Some concerns | No concerns | No concerns | No concerns | High |
| ADF vs Usual | Some concerns | Low risk | Some concerns | No concerns | Some concerns | No concerns | High |
| CER vs PF | Some concerns | High risk | Some concerns | No concerns | No concerns | No concerns | Moderate |
| CER vs TRE | Some concerns | Low risk | Some concerns | No concerns | No concerns | No concerns | High |
| CER vs Usual | Some concerns | Low risk | Some concerns | No concerns | No concerns | No concerns | High |
| CER vs mADF | Some concerns | Some concerns | Some concerns | No concerns | No concerns | No concerns | High |
| PF vs Usual | Some concerns | Low risk | Some concerns | No concerns | No concerns | No concerns | High |
| TRE vs Usual | Some concerns | High risk | Some concerns | No concerns | No concerns | No concerns | Moderate |
| mADF vs Usual | Some concerns | Low risk | Some concerns | No concerns | Some concerns | No concerns | High |
| ADF vs PF | Some concerns | High risk | Some concerns | No concerns | No concerns | Some concerns | Moderate |
| ADF vs mADF | Some concerns | Low risk | Some concerns | No concerns | No concerns | Some concerns | High |
| PF vs TRE | Some concerns | Low risk | Some concerns | No concerns | No concerns | Some concerns | High |
| mADF vs PF | Some concerns | High risk | Some concerns | No concerns | No concerns | Some concerns | Moderate |
| mADF vs TRE | Some concerns | Some concerns | Some concerns | No concerns | No concerns | Some concerns | High |

1. FFM

| Comparison | Within-study bias | Reporting bias | Indirectness | Imprecision | Heterogeneity | Incoherence | Confidence rating |
| --- | --- | --- | --- | --- | --- | --- | --- |
| ADF vs CER | Some concerns | Some concerns | Some concerns | No concerns | No concerns | No concerns | High |
| ADF vs TRE | Some concerns | Some concerns | Some concerns | No concerns | No concerns | No concerns | High |
| ADF vs Usual | Some concerns | Some concerns | Some concerns | No concerns | No concerns | No concerns | High |
| CER vs PF | Some concerns | Low risk | Some concerns | No concerns | No concerns | No concerns | High |
| CER vs TRE | Some concerns | Low risk | Some concerns | No concerns | No concerns | No concerns | High |
| CER vs Usual | Some concerns | Low risk | Some concerns | No concerns | No concerns | No concerns | High |
| CER vs mADF | Some concerns | Some concerns | Some concerns | No concerns | No concerns | No concerns | High |
| PF vs Usual | Some concerns | Low risk | Some concerns | No concerns | No concerns | No concerns | High |
| TRE vs Usual | Major concerns | Low risk | Some concerns | No concerns | No concerns | No concerns | Moderate |
| mADF vs Usual | Some concerns | Some concerns | Some concerns | No concerns | No concerns | No concerns | High |
| ADF vs PF | Some concerns | High risk | Some concerns | No concerns | No concerns | No concerns | Moderate |
| ADF vs mADF | Some concerns | Low risk | Some concerns | No concerns | No concerns | No concerns | High |
| PF vs TRE | Some concerns | Low risk | Some concerns | No concerns | No concerns | No concerns | High |
| mADF vs PF | Some concerns | High risk | Some concerns | No concerns | No concerns | No concerns | Moderate |
| mADF vs TRE | Some concerns | High risk | Some concerns | No concerns | No concerns | No concerns | Moderate |

1. Waist circumference

| Comparison | Within-study bias | Reporting bias | Indirectness | Imprecision | Heterogeneity | Incoherence | Confidence rating |
| --- | --- | --- | --- | --- | --- | --- | --- |
| ADF vs CER | Some concerns | Some concerns | Some concerns | No concerns | No concerns | No concerns | High |
| ADF vs Usual | Some concerns | Low risk | Some concerns | No concerns | No concerns | No concerns | High |
| CER vs PF | Some concerns | Low risk | Some concerns | No concerns | No concerns | No concerns | High |
| CER vs TRE | Some concerns | Low risk | Some concerns | No concerns | No concerns | No concerns | High |
| CER vs Usual | Some concerns | Low risk | Some concerns | No concerns | No concerns | No concerns | High |
| CER vs mADF | Some concerns | Some concerns | Some concerns | No concerns | No concerns | No concerns | High |
| PF vs Usual | Some concerns | Low risk | Some concerns | No concerns | No concerns | No concerns | High |
| TRE vs Usual | Some concerns | Low risk | Some concerns | No concerns | No concerns | No concerns | High |
| mADF vs Usual | Some concerns | Some concerns | Some concerns | No concerns | No concerns | No concerns | High |
| ADF vs PF | Some concerns | High risk | Some concerns | No concerns | No concerns | No concerns | Moderate |
| ADF vs TRE | Some concerns | High risk | Some concerns | No concerns | No concerns | No concerns | Moderate |
| ADF vs mADF | Some concerns | Low risk | Some concerns | No concerns | No concerns | No concerns | High |
| PF vs TRE | Some concerns | Low risk | Some concerns | No concerns | No concerns | No concerns | High |
| mADF vs PF | Some concerns | High risk | Some concerns | No concerns | No concerns | No concerns | Moderate |
| mADF vs TRE | Some concerns | High risk | Some concerns | No concerns | No concerns | No concerns | Moderate |

1. LDL

| Comparison | Within-study bias | Reporting bias | Indirectness | Imprecision | Heterogeneity | Incoherence | Confidence rating |
| --- | --- | --- | --- | --- | --- | --- | --- |
| ADF vs CER | Some concerns | Some concerns | Some concerns | No concerns | No concerns | No concerns | High |
| ADF vs TRE | Some concerns | Some concerns | Some concerns | No concerns | No concerns | No concerns | High |
| ADF vs Usual | Some concerns | Some concerns | Some concerns | No concerns | No concerns | No concerns | High |
| CER vs PF | Some concerns | Some concerns | Some concerns | No concerns | No concerns | No concerns | High |
| CER vs TRE | Some concerns | Low risk | Some concerns | No concerns | No concerns | No concerns | High |
| CER vs Usual | Some concerns | Low risk | Some concerns | No concerns | No concerns | No concerns | High |
| CER vs mADF | Some concerns | Some concerns | Some concerns | No concerns | No concerns | No concerns | High |
| PF vs Usual | Some concerns | Low risk | Some concerns | No concerns | No concerns | No concerns | High |
| TRE vs Usual | Some concerns | Some concerns | Some concerns | No concerns | No concerns | No concerns | High |
| mADF vs Usual | Some concerns | Some concerns | Some concerns | No concerns | No concerns | No concerns | High |
| ADF vs PF | Some concerns | High risk | Some concerns | No concerns | No concerns | No concerns | Moderate |
| ADF vs mADF | Some concerns | Low risk | Some concerns | No concerns | No concerns | No concerns | High |
| PF vs TRE | Some concerns | Low risk | Some concerns | No concerns | No concerns | No concerns | High |
| mADF vs PF | Some concerns | High risk | Some concerns | No concerns | No concerns | No concerns | Moderate |
| mADF vs TRE | Some concerns | High risk | Some concerns | No concerns | No concerns | No concerns | Moderate |

1. SBP

| Comparison | Within-study bias | Reporting bias | Indirectness | Imprecision | Heterogeneity | Incoherence | Confidence rating |
| --- | --- | --- | --- | --- | --- | --- | --- |
| ADF vs CER | Some concerns | Some concerns | Some concerns | No concerns | No concerns | No concerns | High |
| ADF vs Usual | Some concerns | Low risk | Some concerns | No concerns | No concerns | No concerns | High |
| CER vs PF | Some concerns | Low risk | Some concerns | No concerns | No concerns | No concerns | High |
| CER vs TRE | Some concerns | Low risk | Some concerns | No concerns | No concerns | No concerns | High |
| CER vs Usual | Some concerns | Low risk | Some concerns | No concerns | No concerns | No concerns | High |
| CER vs mADF | Some concerns | Some concerns | Some concerns | No concerns | No concerns | No concerns | High |
| PF vs Usual | Some concerns | Low risk | Some concerns | No concerns | No concerns | No concerns | High |
| TRE vs Usual | Some concerns | Low risk | Some concerns | No concerns | No concerns | No concerns | High |
| mADF vs Usual | Some concerns | Low risk | Some concerns | No concerns | No concerns | No concerns | High |
| ADF vs PF | Some concerns | High risk | Some concerns | No concerns | No concerns | No concerns | Moderate |
| ADF vs TRE | Some concerns | High risk | Some concerns | No concerns | No concerns | No concerns | Moderate |
| ADF vs mADF | Some concerns | Low risk | Some concerns | No concerns | No concerns | No concerns | High |
| PF vs TRE | Some concerns | Low risk | Some concerns | No concerns | No concerns | No concerns | High |
| mADF vs PF | Some concerns | High risk | Some concerns | No concerns | No concerns | No concerns | Moderate |
| mADF vs TRE | Some concerns | High risk | Some concerns | No concerns | No concerns | No concerns | Moderate |

1. DBP

| Comparison | Within-study bias | Reporting bias | Indirectness | Imprecision | Heterogeneity | Incoherence | Confidence rating |
| --- | --- | --- | --- | --- | --- | --- | --- |
| ADF vs CER | Some concerns | Some concerns | Some concerns | No concerns | No concerns | No concerns | High |
| ADF vs Usual | Some concerns | Some concerns | Some concerns | No concerns | No concerns | Some concerns | High |
| CER vs PF | Some concerns | Low risk | Some concerns | No concerns | No concerns | No concerns | High |
| CER vs TRE | Some concerns | Low risk | Some concerns | No concerns | No concerns | No concerns | High |
| CER vs Usual | Some concerns | Low risk | Some concerns | No concerns | No concerns | No concerns | High |
| CER vs mADF | Some concerns | Some concerns | Some concerns | No concerns | No concerns | No concerns | High |
| PF vs Usual | Some concerns | Low risk | Some concerns | No concerns | No concerns | No concerns | High |
| TRE vs Usual | Some concerns | Low risk | Some concerns | No concerns | No concerns | No concerns | High |
| mADF vs Usual | Some concerns | Some concerns | Some concerns | No concerns | No concerns | No concerns | High |
| ADF vs PF | Some concerns | High risk | Some concerns | No concerns | No concerns | No concerns | Moderate |
| ADF vs TRE | Some concerns | High risk | Some concerns | No concerns | No concerns | No concerns | Moderate |
| ADF vs mADF | Some concerns | Low risk | Some concerns | No concerns | No concerns | No concerns | High |
| PF vs TRE | Some concerns | Low risk | Some concerns | No concerns | No concerns | No concerns | High |
| mADF vs PF | Some concerns | High risk | Some concerns | No concerns | No concerns | No concerns | Moderate |
| mADF vs TRE | Some concerns | High risk | Some concerns | No concerns | No concerns | No concerns | Moderate |

1. FPG

| Comparison | Within-study bias | Reporting bias | Indirectness | Imprecision | Heterogeneity | Incoherence | Confidence rating |
| --- | --- | --- | --- | --- | --- | --- | --- |
| ADF vs CER | Some concerns | Some concerns | Some concerns | No concerns | No concerns | No concerns | High |
| ADF vs Usual | Major concerns | Some concerns | Some concerns | No concerns | No concerns | No concerns | Moderate |
| CER vs PF | Some concerns | Low risk | Some concerns | No concerns | No concerns | No concerns | High |
| CER vs TRE | Some concerns | Low risk | Some concerns | No concerns | No concerns | No concerns | High |
| CER vs Usual | Some concerns | Low risk | Some concerns | No concerns | No concerns | No concerns | High |
| CER vs mADF | Some concerns | Some concerns | Some concerns | No concerns | No concerns | No concerns | High |
| PF vs Usual | Some concerns | Low risk | Some concerns | No concerns | No concerns | No concerns | High |
| TRE vs Usual | Some concerns | Low risk | Some concerns | No concerns | No concerns | No concerns | High |
| mADF vs Usual | Some concerns | Some concerns | Some concerns | No concerns | No concerns | No concerns | High |
| ADF vs PF | Some concerns | High risk | Some concerns | No concerns | No concerns | No concerns | Moderate |
| ADF vs TRE | Some concerns | High risk | Some concerns | No concerns | No concerns | No concerns | Moderate |
| ADF vs mADF | Some concerns | Low risk | Some concerns | No concerns | No concerns | No concerns | High |
| PF vs TRE | Some concerns | Low risk | Some concerns | No concerns | No concerns | No concerns | High |
| mADF vs PF | Some concerns | High risk | Some concerns | No concerns | No concerns | No concerns | Moderate |
| mADF vs TRE | Some concerns | High risk | Some concerns | No concerns | No concerns | No concerns | Moderate |

ADF-alternate fasting, CER- Continuous energy restrictions, DBP- diastolic blood pressure, FFM- fat-free mass, FPG- fasting plasma glucose, LDL- low density lipoprotein, mADF- modified alternate day fasting, PF- Periodic fasting, SBP- systolic blood pressure, TRE- Time restricted eating

Supplementary Table 2: Classification of intermittent fasting interventions

| Out comes | Certainty on the evidence | Classification | | Intervention | | Intervention vs. Usual care MD (95%CI) | SUCRA |
| --- | --- | --- | --- | --- | --- | --- | --- |
| Body weight reduction (kg) | High Certainty (Moderate- to High-quality evidence | Category 2: among the most effective | | mADF | | -5.18 (-7.04,-3.32) | 90% |
|  |  | Category 1: inferior to the most effective, or superior to the least effective | | ADF | | -4.27 (-6.12,-2.42) | 70% |
|  |  |  |  | PF | | -3.82 (-5.44,-2.21) | 60% |
|  |  |  |  | CER | | -3.42 (-4.73,-2.11) | 50% |
|  |  |  |  | TRE | | -1.93 (-3.06,-0.81) | 20% |
|  |  | Category 0: among the least effective | | - | | - | - |
|  | Low Certainty (Low- to Very low-quality evidence) | Category 2: might be among the most effective | | - | | - | - |
|  |  | Category 1: might be inferior to the most effective or superior than the least effective | | - | | - | - |
|  |  | Category 0: might be among the least effective | | - | | - | - |
|  |  |  | |  | |  |  |
| Fat free mass reduction | Certainty on the evidence | Classification | | Intervention | | Intervention vs. Usual care MD (95%CI) | SUCRA |
|  | High Certainty (Moderate- to High-quality evidence | Category 1: among the most effective | | TRE | | -0.82 (-1.46,-0.17) | 60% |
|  |  |  |  | PF | | -0.80 (-1.58,-0.02) | 60% |
|  |  | Category 0: among the least effective | | mADF | | -1.08 (-2.16,0.01) | 70% |
|  |  |  | | ADF | | -1.01 (-2.07,0.06) | 70 |
|  |  |  | | CER | | -0.63 (-1.30,0.04) | 40 |
|  | Low Certainty (Low- to Very low-quality evidence) | Category 1: might be among the most effective | | - | | - | - |
|  |  | Category 0: might be among the least effective | | - | | - | - |
|  |  |  | |  | |  |  |
| Waist circumference | High Certainty (Moderate- to High-quality evidence | Category 1: among the most effective | | mADF | | -3.55 (-5.66,-1.45) | 80% |
|  |  |  |  | TRE | | -3.00 (-4.50,-1.51) | 70% |
|  |  |  |  | PF | | -2.77 (-4.47,-1.07) | 60% |
|  |  |  |  | CER | | -1.78 (-3.23,-0.34) | 30% |
|  |  | Category 0: among the least effective | | ADF | | -2.86 (-5.88,0.16) | 60% |
|  | Low Certainty (Low- to Very low-quality evidence) | Category 1: might be among the most effective | | - | | - | - |
|  |  | Category 0: might be among the least effective | | - | | - | - |
|  |  |  | |  | |  |  |
| Low density Lipoprotein -LDL | High Certainty (Moderate- to High-quality evidence | Category 1: among the most effective | | PF | | -6.80 (-12.59,-1.00) | 90% |
|  |  | Category 0: among the least effective | | CER | | -3.92 (-8.67,0.84) | 60% |
|  |  |  |  | TRE | | -3.30 (-7.44,0.85) | 60% |
|  |  |  |  | mADF | | -2.96 (-12.13,6.21) | 50% |
|  |  |  |  | ADF | | 0.37 (-8.10,8.83) | 20% |
|  | Low Certainty (Low- to Very low-quality evidence) | Category 1: might be among the most effective | | - | | - | - |
|  |  | Category 0: might be among the least effective | | - | | - | - |
|  |  |  | |  | |  |  |
| Systolic blood pressure -SBP | High Certainty (Moderate- to High-quality evidence | Category 1: among the most effective | | mADF | | -7.24 (-11.90,-2.58) | 90% |
|  |  |  |  | CER | | -4.55 (-6.82,-2.27) | 80% |
|  |  |  |  | PF | | -3.17 (-6.01,-0.32) | 50% |
|  |  |  |  | TRE | | -3.18 (-5.22,-1.13) | 50% |
|  |  | Category 0: among the least effective | | ADF | | -1.17 (-4.61,2.28) | 20% |
|  | Low Certainty (Low- to Very low-quality evidence) | Category 1: might be among the most effective | | - | | - | - |
|  |  | Category 0: might be among the least effective | | - | | - | - |
|  |  |  | |  | |  |  |
| Diastolic blood pressure -DBP | High Certainty (Moderate- to High-quality evidence | Category 1: among the most effective | | mADF | | -4.70 (-8.46,-0.95) | 90% |
|  |  |  |  | TRE | | -3.24 (-4.69,-1.79) | 70% |
|  |  |  |  | PF | | -2.90 (-4.79,-1.02) | 60% |
|  |  |  |  | CER | | -2.66 (-4.11,-1.22) | 50% |
|  |  | Category 0: among the least effective | | ADF | | 0.49 (-1.95,2.93) | 10% |
|  | Low Certainty (Low- to Very low-quality evidence) | Category 1: might be among the most effective | | - | | - | - |
|  |  | Category 0: might be among the least effective | | - | | - | - |
|  |  |  | |  | |  |  |
| Fasting plasma glucose- FPG | Certainty on the evidence | Classification | Intervention | | Intervention vs. Usual care MD (95%CI) | | SUCRA |
|  | High Certainty (Moderate- to High-quality evidence | Category 1: among the most effective | TRE | | -3.74 (-6.01, -1.46) | | 80% |
|  |  | Category 0: among the least effective | mADF | | -4.14 (-8.46,0.18) | | 80% |
|  |  |  | ADF | | -2.74 (-7.37,1.88) | | 60% |
|  |  |  | CER | | -0.28 (-2.87,2.31) | | 30% |
|  |  |  | PF | | -0.12 (-3.17,2.93) | | 20% |
|  | Low Certainty (Low- to Very low-quality evidence) | Category 1: might be among the most effective | - | | - | | - |
|  |  | Category 0: might be among the least effective | - | | - | | - |

Supplementary Table 3: The number of studies each treatment comparison for each outcome

| **Treatment comparison** | **number of studies** |
| --- | --- |
| **Weight** | |
| ADF CER | 2 |
| ADF vs CER vs Usual | 1 |
| ADF vs TRE | 1 |
| ADF vs Usual | 3 |
| CER vs PF | 14 |
| CER vs PF vs Usual | 1 |
| CER vs TRE | 4 |
| CER vs TRE vs Usual | 2 |
| CER vs Usual vs mADF | 1 |
| CER vs mADF | 4 |
| PF vs Usual | 2 |
| TRE vs Usual | 14 |
| Usual vs mADF | 3 |
| Total | 52 |
| **FFM** | |
| ADF vs CER | 2 |
| ADF vs CER vs Usual | 1 |
| ADF vs TRE | 1 |
| ADF vs Usual | 1 |
| CER vs PF | 10 |
| CER vs PF vs Usual | 1 |
| CER vs TRE | 3 |
| CER vs TRE vs Usual | 1 |
| CER vs Usual vs mADF | 1 |
| CER vs mADF | 3 |
| PF vs Usual | 1 |
| TRE vs Usual | 6 |
| Usual vs mADF | 1 |
| Total | 32 |
| **WC** | |
| ADF vs CER | 1 |
| ADF vs Usual | 1 |
| CER vs PF | 7 |
| CER vs PF vs Usual | 1 |
| CER vs TRE | 2 |
| CER vs TRE vs Usual | 2 |
| CER vs mADF | 3 |
| PF vs Usual | 1 |
| TRE vs Usual | 3 |
| Usual vs mADF | 1 |
| Total | 22 |
| **LDL** | |
| ADF vs CER | 1 |
| ADF vs CER vs Usual | 1 |
| ADF vs TRE | 1 |
| ADF vs Usual | 1 |
| CER vs PF | 9 |
| CER vs PF vs Usual | 2 |
| CER vs TRE | 3 |
| CER vs TRE vs Usual | 2 |
| CER vs Usual vs mADF | 1 |
| CER vs mADF | 1 |
| PF vs Usual | 1 |
| TRE vs Usual | 10 |
| Usual vs mADF | 2 |
| Total | 35 |
| **SBP** | |
| ADF vs CER vs Usual | 1 |
| ADF vs Usual | 2 |
| CER vs PF | 7 |
| CER vs PF vs Usual | 1 |
| CER vs TRE | 3 |
| CER vs TRE vs Usual | 2 |
| CER vs Usual vs mADF | 1 |
| CER vs mADF | 1 |
| PF vs Usual | 1 |
| TRE vs Usual | 6 |
| Usual vs mADF | 2 |
| Total | 27 |
| **DBP** | |
| ADF vs CER vs Usual | 1 |
| ADF vs Usual | 2 |
| CER vs PF | 7 |
| CER vs PF vs Usual | 1 |
| CER vs TRE | 3 |
| CER vs TRE vs Usual | 2 |
| CER vs Usual vs mADF | 1 |
| CER vs mADF | 1 |
| PF vs Usual | 1 |
| TRE vs Usual | 6 |
| Usual vs mADF | 2 |
| Total | 27 |
| **FPG** | |
| ADF vs CER | 1 |
| ADF vs CER vs Usual | 1 |
| ADF vs Usual | 2 |
| CER vs PF | 9 |
| CER vs PF vs Usual | 2 |
| CER vs TRE | 4 |
| CER vs TRE vs Usual | 1 |
| CER vs Usual vs mADF | 1 |
| CER vs mADF | 2 |
| PF vs Usual | 1 |
| TRE vs Usual | 10 |
| Usual vs mADF | 2 |
| Total | 36 |

ADF-alternate fasting, CER- Continuous energy restrictions, DBP- diastolic blood pressure, FFM- fat-free mass, FPG- fasting plasma glucose, LDL- low density lipoprotein, mADF- modified alternate day fasting, PF- Periodic fasting, SBP- systolic blood pressure, TRE- Time restricted eating

Supplementary Table 4: Local inconsistency assessment

| **Side /Comparison** | **Direct** | | **Indirect** | | **Difference** | | **P-value** | **Tau** |
| --- | --- | --- | --- | --- | --- | --- | --- | --- |
|  | **Coef.** | **Std. Err.** | **Coef.** | **Std. Err.** | **Coef.** | **Std. Err.** |  |  |
| **Weight** | | | | | | | | |
| Usual vs mADF | -4.126 | 1.228 | -6.611 | 1.431 | 2.485 | 1.875 | 0.185 | 2.033 |
| ADF vs Usual | 4.599 | 1.132 | 3.487 | 1.740 | 1.112 | 2.075 | 0.592 | 2.081 |
| ADF vs CER | 0.184 | 1.725 | 1.248 | 1.330 | -1.064 | 2.178 | 0.625 | 2.085 |
| ADF vs TRE | 2.500 | 2.265 | 2.290 | 1.164 | 0.210 | 2.547 | 0.934 | 2.090 |
| CER vs Usual | 4.530 | 1.038 | 2.656 | 0.865 | 1.873 | 1.347 | 0.164 | 2.053 |
| CER vs PF | -0.384 | 0.714 | -0.531 | 1.712 | 0.147 | 1.855 | 0.937 | 2.091 |
| CER vs TRE | 1.160 | 0.997 | 1.859 | 1.078 | -0.699 | 1.468 | 0.634 | 2.092 |
| CER vs mADF | -2.837 | 1.141 | 0.163 | 1.508 | -3.000 | 1.878 | 0.110 | 2.020 |
| PF vs Usual | 4.021 | 1.286 | 3.680 | 1.088 | 0.341 | 1.684 | 0.840 | 2.091 |
| TRE vs Usual | 2.001 | 0.649 | 1.677 | 1.284 | 0.323 | 1.438 | 0.822 | 2.085 |
| **FFM** | | | | | | | | |
| Usual vs mADF | -1.282 | 0.856 | -0.925 | 0.734 | -0.357 | 1.128 | 0.752 | 0.707 |
| ADF vs Usual | 0.819 | 0.724 | 1.267 | 0.850 | -0.448 | 1.115 | 0.688 | 0.714 |
| ADF vs CER | -0.689 | 0.738 | 1.670 | 0.807 | -2.359 | 1.124 | 0.036 | 0.613 |
| ADF vs TRE | 1.800 | 1.004 | -0.495 | 0.652 | 2.295 | 1.198 | 0.055 | 0.658 |
| CER vs Usual | 1.252 | 0.482 | 0.093 | 0.439 | 1.159 | 0.649 | 0.074 | 0.639 |
| CER vs PF | -0.163 | 0.324 | -0.197 | 0.863 | 0.034 | 0.922 | 0.971 | 0.719 |
| CER vs TRE | -0.787 | 0.401 | 0.817 | 0.546 | -1.604 | 0.675 | 0.017 | 0.614 |
| CER vs mADF | -0.320 | 0.594 | -0.823 | 1.040 | 0.503 | 1.197 | 0.674 | 0.707 |
| PF vs Usual | 0.939 | 0.612 | 0.696 | 0.538 | 0.243 | 0.812 | 0.765 | 0.715 |
| TRE vs Usual | 0.547 | 0.372 | 1.529 | 0.602 | -0.982 | 0.707 | 0.165 | 0.653 |
| **WC** | | | | | | | | |
| Usual vs mADF | -0.700 | 1.544 | -5.308 | 1.211 | 4.608 | 1.963 | 0.019 | 1.014 |
| ADF vs Usual | 4.000 | 1.919 | 0.807 | 2.577 | 3.193 | 3.213 | 0.320 | 1.297 |
| ADF vs CER | -0.800 | 2.463 | 2.393 | 2.063 | -3.193 | 3.213 | 0.320 | 1.297 |
| CER vs Usual | 3.729 | 0.905 | 0.138 | 0.817 | 3.590 | 1.219 | 0.003 | 0.827 |
| CER vs PF | -0.921 | 0.690 | -1.547 | 2.042 | 0.625 | 2.155 | 0.772 | 1.350 |
| CER vs TRE | -1.593 | 0.870 | 0.107 | 1.622 | -1.700 | 1.842 | 0.356 | 1.327 |
| CER vs mADF | -2.951 | 0.987 | 1.657 | 1.697 | -4.608 | 1.963 | 0.019 | 1.014 |
| PF vs Usual | 3.235 | 1.330 | 2.407 | 1.184 | 0.828 | 1.777 | 0.641 | 1.350 |
| TRE vs Usual | 2.775 | 0.910 | 3.596 | 1.527 | -0.822 | 1.777 | 0.644 | 1.366 |
| **LDL** | | | | | | | | |
| Usual vs mADF | -2.079 | 5.985 | -4.553 | 7.778 | 2.474 | 9.809 | 0.801 | 6.334 |
| ADF vs Usual | 2.672 | 5.373 | -5.461 | 6.985 | 8.134 | 8.753 | 0.353 | 6.063 |
| ADF vs CER | -4.605 | 5.921 | -3.842 | 6.999 | -0.763 | 9.239 | 0.934 | 6.355 |
| ADF vs TRE | -14.400 | 10.358 | -1.141 | 5.046 | -13.259 | 11.522 | 0.250 | 6.205 |
| CER vs Usual | 4.348 | 3.267 | 3.392 | 3.807 | 0.956 | 5.029 | 0.849 | 6.349 |
| CER vs PF | -2.221 | 2.336 | -10.099 | 7.860 | 7.879 | 8.225 | 0.338 | 6.329 |
| CER vs TRE | -1.095 | 3.483 | 2.836 | 3.938 | -3.931 | 5.263 | 0.455 | 6.199 |
| CER vs mADF | 3.543 | 5.834 | -3.875 | 7.948 | 7.418 | 9.863 | 0.452 | 6.291 |
| PF vs Usual | 9.069 | 4.806 | 5.266 | 3.944 | 3.803 | 6.236 | 0.542 | 6.425 |
| TRE vs Usual | 1.720 | 2.278 | 10.137 | 4.751 | -8.417 | 5.271 | 0.110 | 5.920 |
| **SBP** | | | | | | | | |
| Usual vs mADF | -5.229 | 2.601 | -15.094 | 4.982 | 9.865 | 5.506 | 0.073 | 2.148 |
| ADF vs Usual | 1.784 | 1.795 | -6.128 | 5.495 | 7.911 | 5.651 | 0.162 | 2.182 |
| ADF vs CER | -7.003 | 2.892 | -0.599 | 2.546 | -6.405 | 3.830 | 0.094 | 2.107 |
| CER vs Usual | 5.499 | 1.607 | 3.493 | 1.686 | 2.006 | 2.304 | 0.384 | 2.278 |
| CER vs PF | 1.262 | 1.099 | 2.963 | 4.102 | -1.701 | 4.250 | 0.689 | 2.274 |
| CER vs TRE | 1.108 | 1.405 | 1.963 | 2.077 | -0.855 | 2.515 | 0.734 | 2.276 |
| CER vs mADF | -9.101 | 4.460 | 0.122 | 2.963 | -9.222 | 5.299 | 0.082 | 2.146 |
| PF vs Usual | 2.914 | 2.592 | 3.281 | 1.794 | -0.367 | 3.149 | 0.907 | 2.295 |
| TRE vs Usual | 3.004 | 1.178 | 3.769 | 2.330 | -0.765 | 2.598 | 0.768 | 2.278 |
| **DBP** | | | | | | | | |
| Usual vs mADF | -3.366 | 2.481 | -6.407 | 2.780 | 3.041 | 3.596 | 0.398 | 1.381 |
| ADF vs Usual | 0.048 | 1.311 | -4.540 | 3.264 | 4.588 | 3.433 | 0.181 | 1.387 |
| ADF vs CER | -4.971 | 1.669 | -0.859 | 1.912 | -4.113 | 2.479 | 0.097 | 1.314 |
| CER vs Usual | 2.586 | 0.985 | 2.709 | 1.148 | -0.123 | 1.492 | 0.935 | 1.466 |
| CER vs PF | -0.542 | 0.688 | 3.311 | 2.448 | -3.853 | 2.538 | 0.129 | 1.314 |
| CER vs TRE | -0.393 | 0.918 | -0.977 | 1.393 | 0.584 | 1.671 | 0.727 | 1.469 |
| CER vs mADF | -3.297 | 2.527 | -0.499 | 2.782 | -2.799 | 3.657 | 0.444 | 1.382 |
| PF vs Usual | 1.728 | 1.571 | 3.528 | 1.172 | -1.800 | 1.912 | 0.347 | 1.395 |
| TRE vs Usual | 3.430 | 0.871 | 2.548 | 1.502 | 0.882 | 1.711 | 0.606 | 1.481 |
| **FPG** | | | | | | | | |
| Usual vs Madf | -3.922 | 3.354 | -4.319 | 3.015 | 0.397 | 4.539 | 0.930 | 3.654 |
| ADF vs Usual | 4.928 | 2.843 | -1.580 | 3.964 | 6.509 | 4.835 | 0.178 | 3.533 |
| ADF vs CER | 0.959 | 2.863 | 5.560 | 4.092 | -4.601 | 4.965 | 0.354 | 3.594 |
| CER vs Usual | 1.550 | 1.881 | -0.926 | 1.835 | 2.475 | 2.615 | 0.344 | 3.594 |
| CER vs PF | 0.250 | 1.304 | -0.565 | 3.694 | 0.815 | 3.919 | 0.835 | 3.667 |
| CER vs TRE | -5.465 | 2.049 | -1.491 | 2.036 | -3.974 | 2.881 | 0.168 | 3.500 |
| CER vs mADF | -3.665 | 2.400 | -4.396 | 4.072 | 0.731 | 4.704 | 0.876 | 3.655 |
| PF vs Usual | 0.308 | 2.219 | -0.045 | 2.253 | 0.353 | 3.161 | 0.911 | 3.669 |
| TRE vs Usual | 2.965 | 1.243 | 7.455 | 2.733 | -4.490 | 3.003 | 0.135 | 3.471 |

ADF-alternate fasting, CER- Continuous energy restrictions, DBP- diastolic blood pressure, FFM- fat-free mass, FPG- fasting plasma glucose, LDL- low density lipoprotein, mADF- modified alternate day fasting, PF- Periodic fasting, SBP- systolic blood pressure, TRE- Time restricted eating
