# Supplementary material for: Intermittent Fasting for the Prevention of Cardiovascular Disease Risks: Systematic Review and Network Meta-Analysis
Source: Curr Nutr Rep. 2025 Jul 24;14(1):93. doi: 10.1007/s13668-025-00684-7 (PMC12289860; doi:10.1007/s13668-025-00684-7)
Supplement: Supplementary file 4 — Supplementary Material 4 [file 13668_2025_684_MOESM4_ESM.docx]

**Intermittent fasting for the prevention of cardiovascular disease risks: systematic review and network meta-analysis**

**Supplementary Figures**

[Supplement Figure 1: Risk of bias for each study 2](#_Toc189056016)

[Supplement Figure 2 : Global inconsistency 10](#_Toc189056017)

[Supplement Figure 3: Intervention rankings based on SUCRA 16](#_Toc189056018)

[Supplement Figure 4: Sensitivity analysis of network meta-analysis pooled estimates (mean difference with 95% CI ) excluding studies that included T2DM patients. Weight in kg (A); Fat-free mass in kg(B); Waist circumference in cm (C); Low density lipoprotein-LDL in mg/dl (D); Systolic blood pressure -SBP in mmHg(E); Diastolic blood pressure - DBP in mmHg (F); Fasting plasma glucose – FPG in mg/dl (G). Values in bold indicate a statistically significant effect compared to usual diet. 19](#_Toc189056019)

[Supplement Figure 5: Sensitivity analysis of network meta-analysis pooled estimates (mean difference with 95% CI ) excluding studies with high-risk of bias: Weight in kg (A); Fat-free mass in kg(B); Waist circumference in cm (C); Low density lipoprotein-LDL in mg/dl (D); Systolic blood pressure -SBP in mmHg(E); Diastolic blood pressure - DBP in mmHg (F); Fasting plasma glucose – FPG in mg/dl (G). Values in bold indicate a statistically significant effect compared to usual diet. 22](#_Toc189056020)

[Supplement Figure 6: Sensitivity analysis of network meta-analysis pooled estimates (mean difference with 95% CI) with medium or long follow up durations (>=3 months). Weight in kg (A); Fat-free mass in kg(B); Waist circumference in cm (C); Low density lipoprotein-LDL in mg/dl (D); Systolic blood pressure -SBP in mmHg(E); Diastolic blood pressure - DBP in mmHg (F); Fasting plasma glucose – FPG in mg/dl (G). Values in bold indicate a statistically significant effect compared to usual diet. 25](#_Toc189056021)


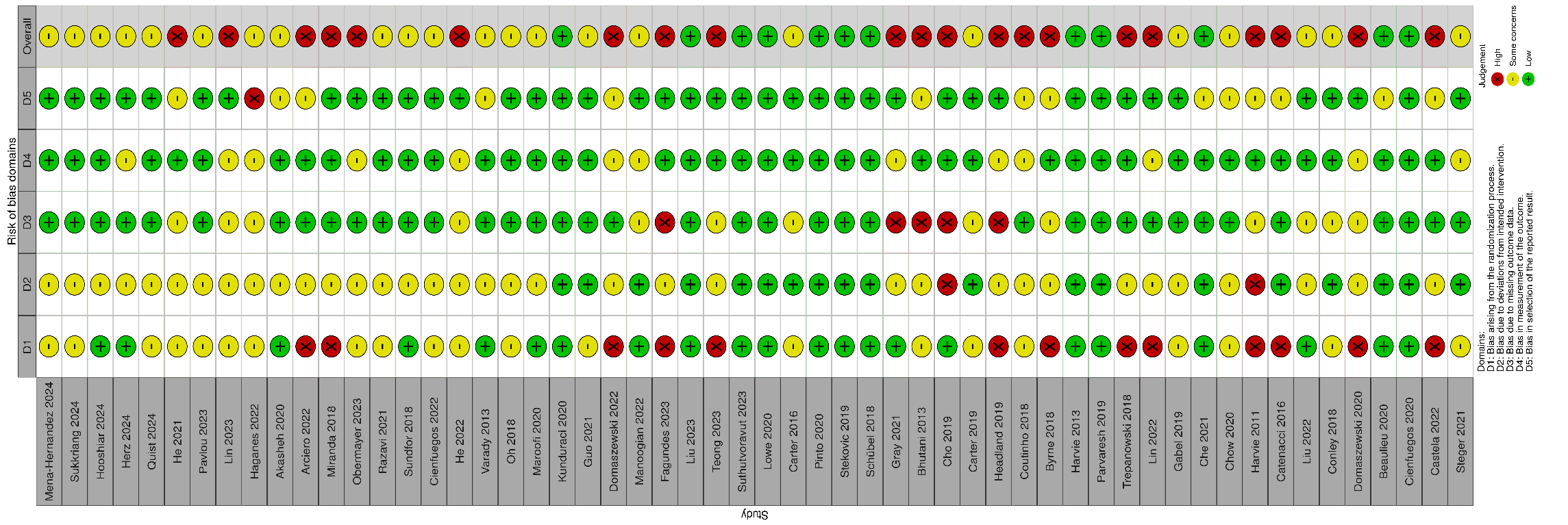


Supplement Figure 1: Risk of bias for each study

Supplement Figure 2 : Global inconsistency

 ****

Supplement Figure 3: Intervention rankings based on SUCRA

|  | Treatment | | | | | |
| --- | --- | --- | --- | --- | --- | --- |
|  | Usual diet | mADF | TRE | PF | CER | ADF |
| Comparator | Usual diet | **-5.65 (-7.66,-3.63)** | **-1.53 (-2.92,-0.14)** | **-4.66 (-7.10,-2.21)** | **-4.28 (-6.08,-2.47)** | **-4.40 (-6.33,-2.46)** |
|  | 5.65 (3.63,7.66) | mADF | 4.12 (1.84,6.40) | 0.99 (-1.56,3.55) | 1.37 (-0.59,3.33) | 1.25 (-1.37,3.87) |
|  | 1.53 (0.14,2.92) | **-4.12 (-6.40,-1.84)** | TRE | -3.13 (-5.66,-0.59) | -2.75 (-4.68,-0.82) | -2.87 (-5.04,-0.70) |
|  | 4.66 (2.21,7.10) | -0.99 (-3.55,1.56) | 3.13 (0.59,5.66) | PF | 0.38 (-1.26,2.02) | 0.26 (-2.57,3.08) |
|  | 4.28 (2.47,6.08) | -1.37 (-3.33,0.59) | 2.75 (0.82,4.68) | -0.38 (-2.02,1.26) | CER | -0.12 (-2.43,2.18) |
|  | 4.40 (2.46,6.33) | -1.25 (-3.87,1.37) | 2.87 (0.70,5.04) | -0.26 (-3.08,2.57) | 0.12 (-2.18,2.43) | ADF |

1. Weight

|  | Treatment | | | | | |
| --- | --- | --- | --- | --- | --- | --- |
|  | Usual diet | mADF | TRE | PF | CER | ADF |
| Comparator | Usual diet | -1.11 (-2.31,0.09) | **-0.87 (-1.62,-0.12)** | -0.64 (-1.88,0.60) | -0.69 (-1.57,0.19) | -1.03 (-2.20,0.13) |
|  | 1.11 (-0.09,2.31) | mADF | 0.24 (-1.00,1.48) | 0.47 (-0.92,1.85) | 0.42 (-0.66,1.49) | 0.07 (-1.45,1.60) |
|  | 0.87 (0.12,1.62) | -0.24 (-1.48,1.00) | TRE | 0.23 (-0.98,1.43) | 0.18 (-0.65,1.00) | -0.16 (-1.36,1.03) |
|  | 0.64 (-0.60,1.88) | -0.47 (-1.85,0.92) | -0.23 (-1.43,0.98) | PF | -0.05 (-0.93,0.83) | -0.39 (-1.88,1.10) |
|  | 0.69 (-0.19,1.57) | -0.42 (-1.49,0.66) | -0.18 (-1.00,0.65) | 0.05 (-0.83,0.93) | CER | -0.34 (-1.55,0.87) |
|  | 1.03 (-0.13,2.20) | -0.07 (-1.60,1.45) | 0.16 (-1.03,1.36) | 0.39 (-1.10,1.88) | 0.34 (-0.87,1.55) | ADF |

1. ffm

|  | Treatment | | | | | |
| --- | --- | --- | --- | --- | --- | --- |
|  | Usual diet | mADF | TRE | PF | CER | ADF |
| Comparator | Usual diet | -2.62 (-5.27,0.03) | **-2.37 (-4.36,-0.38)** | -1.56 (-4.62,1.50) | -0.48 (-2.94,1.97) | -2.32 (-5.61,0.98) |
|  | 2.62 (-0.03,5.27) | mADF | 0.25 (-2.62,3.12) | 1.07 (-1.73,3.86) | 2.14 (0.02,4.25) | 0.31 (-3.51,4.13) |
|  | 2.37 (0.38,4.36) | -0.25 (-3.12,2.62) | TRE | 0.81 (-2.26,3.89) | 1.89 (-0.59,4.36) | 0.06 (-3.59,3.70) |
|  | 1.56 (-1.50,4.62) | -1.07 (-3.86,1.73) | -0.81 (-3.89,2.26) | PF | 1.07 (-0.76,2.91) | -0.76 (-4.71,3.19) |
|  | 0.48 (-1.97,2.94) | -2.14 (-4.25,-0.02) | -1.89 (-4.36,0.59) | -1.07 (-2.91,0.76) | CER | -1.83 (-5.34,1.68) |
|  | 2.32 (-0.98,5.61) | -0.31 (-4.13,3.51) | -0.06 (-3.70,3.59) | 0.76 (-3.19,4.71) | 1.83 (-1.68,5.34) | ADF |

1. wc

|  | Treatment | | | | | |
| --- | --- | --- | --- | --- | --- | --- |
|  | Usual diet | mADF | TRE | PF | CER | ADF |
| Comparator | Usual diet | -2.25 (-10.58,6.08) | -1.52 (-5.67,2.62) | -5.76 (-11.98,0.46) | -2.93 (-8.01,2.15) | 1.07 (-6.29,8.44) |
|  | 2.25 (-6.08,10.58) | Madf | 0.73 (-8.32,9.78) | -3.51 (-12.62,5.60) | -0.68 (-8.94,7.58) | 3.33 (-7.28,13.93) |
|  | 1.52 (-2.62,5.67) | -0.73 (-9.78,8.32) | TRE | -4.24 (-11.03,2.56) | -1.41 (-7.18,4.37) | 2.60 (-5.34,10.53) |
|  | 5.76 (-0.46,11.98) | 3.51 (-5.60,12.62) | 4.24 (-2.56,11.03) | PF | 2.83 (-1.17,6.83) | 6.83 (-1.69,15.36) |
|  | 2.93 (-2.15,8.01) | 0.68 (-7.58,8.94) | 1.41 (-4.37,7.18) | -2.83 (-6.83,1.17) | CER | 4.00 (-3.65,11.66) |
|  | -1.07 (-8.44,6.29) | -3.33 (-13.93,7.28) | -2.60 (-10.53,5.34) | -6.83 (-15.36,1.69) | -4.00 (-11.66,3.65) | ADF |

1. LDL

|  | Treatment | | | | | |
| --- | --- | --- | --- | --- | --- | --- |
|  | Usual diet | mADF | TRE | PF | CER | ADF |
| Comparator | Usual diet | **-7.38 (-12.29,-2.46)** | **-3.38 (-6.12,-0.65)** | **-3.29 (-7.57,0.99)** | **-4.62 (-8.07,-1.17)** | -1.17 (-4.96,2.62) |
|  | 7.38 (2.46,12.29) | mADF | 3.99 (-1.53,9.52) | 4.09 (-2.00,10.18) | 2.76 (-2.79,8.30) | 6.20 (0.08,12.33) |
|  | 3.38 (0.65,6.12) | -3.99 (-9.52,1.53) | TRE | 0.10 (-4.36,4.55) | -1.24 (-4.88,2.41) | 2.21 (-2.33,6.75) |
|  | 3.29 (-0.99,7.57) | -4.09 (-10.18,2.00) | -0.10 (-4.55,4.36) | PF | -1.33 (-3.89,1.23) | 2.12 (-3.17,7.40) |
|  | 4.62 (1.17,8.07) | -2.76 (-8.30,2.79) | 1.24 (-2.41,4.88) | 1.33 (-1.23,3.89) | CER | 3.45 (-1.19,8.08) |
|  | 1.17 (-2.62,4.96) | -6.20 (-12.33,-0.08) | -2.21 (-6.75,2.33) | -2.12 (-7.40,3.17) | -3.45 (-8.08,1.19) | ADF |

1. SBP

|  | Treatment | | | | | |
| --- | --- | --- | --- | --- | --- | --- |
|  | Usual diet | mADF | TRE | PF | CER | ADF |
| Comparator | Usual diet | **-5.02 (-8.64,-1.40)** | **-4.58 (-6.20,-2.96)** | **-3.97 (-6.30,-1.65)** | **-3.31 (-5.08,-1.53)** | 0.42 (-1.73,2.57) |
|  | 5.02 (1.40,8.64) | mADF | 0.44 (-3.40,4.27) | 1.04 (-2.84,4.93) | 1.71 (-1.91,5.33) | 5.44 (1.33,9.54) |
|  | 4.58 (2.96,6.20) | -0.44 (-4.27,3.40) | TRE | 0.61 (-1.73,2.94) | 1.27 (-0.66,3.20) | 5.00 (2.37,7.63) |
|  | 3.97 (1.65,6.30) | -1.04 (-4.93,2.84) | -0.61 (-2.94,1.73) | PF | 0.67 (-0.65,1.98) | 4.39 (1.49,7.30) |
|  | 3.31 (1.53,5.08) | -1.71 (-5.33,1.91) | -1.27 (-3.20,0.66) | -0.67 (-1.98,0.65) | CER | 3.73 (1.27,6.18) |
|  | -0.42 (-2.57,1.73) | -5.44 (-9.54,-1.33) | -5.00 (-7.63,-2.37) | -4.39 (-7.30,-1.49) | -3.73 (-6.18,-1.27) | ADF |

1. DBP

|  | Treatment | | | | | |
| --- | --- | --- | --- | --- | --- | --- |
|  | Usual diet | mADF | TRE | PF | CER | ADF |
| Comparator | Usual diet | -4.08 (-8.68,0.52) | **-3.42 (-6.04,-0.80)** | 0.52 (-3.52,4.55) | -0.21 (-3.39,2.98) | -2.72 (-7.55,2.12) |
|  | 4.08 (-0.52,8.68) | Madf | 0.66 (-4.33,5.65) | 4.60 (-0.39,9.58) | 3.87 (-0.32,8.06) | 1.36 (-4.78,7.50) |
|  | 3.42 (0.80,6.04) | -0.66 (-5.65,4.33) | TRE | 3.94 (-0.43,8.30) | 3.21 (-0.32,6.74) | 0.70 (-4.59,6.00) |
|  | -0.52 (-4.55,3.52) | -4.60 (-9.58,0.39) | -3.94 (-8.30,0.43) | PF | -0.72 (-3.53,2.08) | -3.23 (-8.75,2.28) |
|  | 0.21 (-2.98,3.39) | -3.87 (-8.06,0.32) | -3.21 (-6.74,0.32) | 0.72 (-2.08,3.53) | CER | -2.51 (-7.35,2.33) |
|  | 2.72 (-2.12,7.55) | -1.36 (-7.50,4.78) | -0.70 (-6.00,4.59) | 3.23 (-2.28,8.75) | 2.51 (-2.33,7.35) | ADF |

1. FPG

Supplement Figure 4: Sensitivity analysis of network meta-analysis pooled estimates (mean difference with 95% CI ) excluding studies that included type 2 diabetes patients. Weight in kg (A); Fat-free mass in kg(B); Waist circumference in cm (C); Low density lipoprotein-LDL in mg/dl (D); Systolic blood pressure -SBP in mmHg(E); Diastolic blood pressure - DBP in mmHg (F); Fasting plasma glucose – FPG in mg/dl (G). Values in bold indicate a statistically significant effect compared to usual diet.

|  | Treatment | | | | | |
| --- | --- | --- | --- | --- | --- | --- |
|  | Usual diet | mADF | TRE | PF | CER | ADF |
| Comparator | Usual diet | **-4.36 (-5.99,-2.74)** | **-2.15 (-3.05,-1.25)** | **-2.46 (-4.10,-0.82)** | **-2.54 (-3.77,-1.31)** | **-3.98 (-5.51,-2.45)** |
|  | 4.36 (2.74,5.99) | Madf | 2.21 (0.43,4.00) | 1.90 (-0.11,3.92) | 1.82 (0.26,3.39) | 0.39 (-1.73,2.50) |
|  | 2.15 (1.25,3.05) | **-2.21 (-4.00,-0.43)** | TRE | -0.31 (-2.09,1.47) | -0.39 (-1.76,0.98) | -1.83 (-3.46,-0.20) |
|  | 2.46 (0.82,4.10) | -1.90 (-3.92,0.11) | 0.31 (-1.47,2.09) | PF | -0.08 (-1.46,1.31) | -1.52 (-3.60,0.57) |
|  | 2.54 (1.31,3.77) | -1.82 (-3.39,-0.26) | 0.39 (-0.98,1.76) | 0.08 (-1.31,1.46) | CER | -1.44 (-3.16,0.28) |
|  | 3.98 (2.45,5.51) | -0.39 (-2.50,1.73) | 1.83 (0.20,3.46) | 1.52 (-0.57,3.60) | 1.44 (-0.28,3.16) | ADF |

a.weight

|  | Treatment | | | | | |
| --- | --- | --- | --- | --- | --- | --- |
|  | Usual diet | mADF | TRE | PF | CER | ADF |
| Comparator | Usual diet | -1.16 (-2.34,0.02) | -0.65 (-1.38,0.09) | **-1.03 (-1.94,-0.12)** | **-0.96 (-1.77,-0.15)** | -1.18 (-2.23,-0.12) |
|  | 1.16 (-0.02,2.34) | Madf | 0.51 (-0.80,1.82) | 0.13 (-1.18,1.44) | 0.20 (-0.96,1.36) | -0.02 (-1.51,1.48) |
|  | 0.65 (-0.09,1.38) | **-0.51 (-1.82,0.80)** | TRE | -0.38 (-1.42,0.65) | -0.31 (-1.19,0.57) | -0.53 (-1.64,0.58) |
|  | 1.03 (0.12,1.94) | -0.13 (-1.44,1.18) | 0.38 (-0.65,1.42) | PF | 0.07 (-0.67,0.81) | -0.15 (-1.40,1.10) |
|  | 0.96 (0.15,1.77) | -0.20 (-1.36,0.96) | 0.31 (-0.57,1.19) | -0.07 (-0.81,0.67) | CER | -0.22 (-1.33,0.90) |
|  | 1.18 (0.12,2.23) | 0.02 (-1.48,1.51) | 0.53 (-0.58,1.64) | 0.15 (-1.10,1.40) | 0.22 (-0.90,1.33) | ADF |

b.ffm

|  | Treatment | | | | | |
| --- | --- | --- | --- | --- | --- | --- |
|  | Usual diet | mADF | TRE | PF | CER | ADF |
| Comparator | Usual diet | **-2.95 (-5.15,-0.76)** | **-2.30 (-4.48,-0.12)** | -1.15 (-3.33,1.04) | -0.93 (-2.85,0.99) | -0.13 (-5.18,4.92) |
|  | 2.95 (0.76,5.15) | mADF | 0.66 (-1.95,3.26) | 1.80 (-0.48,4.09) | 2.02 (0.22,3.83) | 2.82 (-2.19,7.83) |
|  | 2.30 (0.12,4.48) | -0.66 (-3.26,1.95) | TRE | 1.15 (-1.35,3.65) | 1.37 (-0.76,3.50) | 2.17 (-2.97,7.30) |
|  | 1.15 (-1.04,3.33) | -1.80 (-4.09,0.48) | -1.15 (-3.65,1.35) | PF | 0.22 (-1.30,1.74) | 1.02 (-3.90,5.93) |
|  | 0.93 (-0.99,2.85) | -2.02 (-3.83,-0.22) | -1.37 (-3.50,0.76) | -0.22 (-1.74,1.30) | CER | 0.80 (-3.87,5.47) |
|  | 0.13 (-4.92,5.18) | -2.82 (-7.83,2.19) | -2.17 (-7.30,2.97) | -1.02 (-5.93,3.90) | -0.80 (-5.47,3.87) | ADF |

c.wc

|  | Treatment | | | | | |
| --- | --- | --- | --- | --- | --- | --- |
|  | Usual diet | mADF | TRE | PF | CER | ADF |
| Comparator | Usual diet | -2.60 (-14.71,9.51) | -2.73 (-8.74,3.29) | -4.38 (-14.11,5.35) | -5.08 (-12.61,2.46) | 3.79 (-9.72,17.29) |
|  | 2.60 (-9.51,14.71) | Madf | -0.13 (-13.12,12.86) | -1.78 (-15.80,12.23) | -2.48 (-14.43,9.47) | 6.38 (-11.07,23.83) |
|  | 2.73 (-3.29,8.74) | 0.13 (-12.86,13.12) | TRE | -1.66 (-12.22,8.91) | -2.35 (-10.52,5.82) | 6.51 (-7.21,20.24) |
|  | 4.38 (-5.35,14.11) | 1.78 (-12.23,15.80) | 1.66 (-8.91,12.22) | PF | -0.69 (-8.90,7.51) | 8.17 (-7.38,23.72) |
|  | 5.08 (-2.46,12.61) | 2.48 (-9.47,14.43) | 2.35 (-5.82,10.52) | 0.69 (-7.51,8.90) | CER | 8.86 (-5.02,22.75) |
|  | -3.79 (-17.29,9.72) | -6.38 (-23.83,11.07) | -6.51 (-20.24,7.21) | -8.17 (-23.72,7.38) | -8.86 (-22.75,5.02) | ADF |

d.LDL

|  | Treatment | | | | | |
| --- | --- | --- | --- | --- | --- | --- |
|  | Usual diet | mADF | TRE | PF | CER | ADF |
| Comparator | Usual diet | **-4.72 (-9.27,-0.17)** | **-3.55 (-5.45,-1.66)** | **-2.91 (-5.15,-0.67)** | **-4.40 (-6.26,-2.53)** | 0.41 (-2.86,3.68) |
|  | 4.72 (0.17,9.27) | mADF | 1.17 (-3.76,6.09) | 1.81 (-3.24,6.86) | 0.32 (-4.57,5.22) | 5.13 (-0.47,10.73) |
|  | 3.55 (1.66,5.45) | **-1.17 (-6.09,3.76)** | TRE | 0.64 (-1.56,2.84) | -0.84 (-2.53,0.85) | 3.96 (0.33,7.59) |
|  | 2.91 (0.67,5.15) | -1.81 (-6.86,3.24) | -0.64 (-2.84,1.56) | PF | -1.49 (-2.95,-0.02) | 3.32 (-0.45,7.09) |
|  | 4.40 (2.53,6.26) | -0.32 (-5.22,4.57) | 0.84 (-0.85,2.53) | 1.49 (0.02,2.95) | CER | 4.80 (1.27,8.34) |
|  | -0.41 (-3.68,2.86) | -5.13 (-10.73,0.47) | -3.96 (-7.59,-0.33) | -3.32 (-7.09,0.45) | -4.80 (-8.34,-1.27) | ADF |

e.SBP

|  | Treatment | | | | | |
| --- | --- | --- | --- | --- | --- | --- |
|  | Usual diet | mADF | TRE | PF | CER | ADF |
| Comparator | Usual diet | -3.87 (-8.67,0.94) | **-2.99 (-4.53,-1.45)** | **-2.57 (-4.39,-0.74)** | **-2.18 (-3.56,-0.81)** | 1.62 (-0.88,4.12) |
|  | 3.87 (-0.94,8.67) | mADF | 0.88 (-4.16,5.91) | 1.30 (-3.83,6.43) | 1.68 (-3.30,6.66) | 5.49 (0.08,10.90) |
|  | 2.99 (1.45,4.53) | **-0.88 (-5.91,4.16)** | TRE | 0.43 (-1.45,2.30) | 0.81 (-0.60,2.21) | 4.61 (1.84,7.39) |
|  | 2.57 (0.74,4.39) | -1.30 (-6.43,3.83) | -0.43 (-2.30,1.45) | PF | 0.38 (-0.90,1.66) | 4.19 (1.31,7.06) |
|  | 2.18 (0.81,3.56) | -1.68 (-6.66,3.30) | -0.81 (-2.21,0.60) | -0.38 (-1.66,0.90) | CER | 3.81 (1.23,6.38) |
|  | -1.62 (-4.12,0.88) | -5.49 (-10.90,-0.08) | -4.61 (-7.39,-1.84) | -4.19 (-7.06,-1.31) | -3.81 (-6.38,-1.23) | ADF |

f.DBP

|  | Treatment | | | | | |
| --- | --- | --- | --- | --- | --- | --- |
|  | Usual diet | mADF | TRE | PF | CER | ADF |
| Comparator | ref | -4.19 (-9.76,1.38) | **-3.50 (-6.20,-0.80)** | -0.54 (-4.72,3.64) | -1.77 (-5.38,1.85) | -5.37 (-12.36,1.63) |
|  | 4.19 (-1.38,9.76) | _y_mADF | 0.69 (-5.35,6.73) | 3.65 (-2.53,9.84) | 2.42 (-2.88,7.73) | -1.18 (-9.68,7.32) |
|  | 3.50 (0.80,6.20) | **-0.69 (-6.73,5.35)** | _y_TRE | 2.97 (-1.82,7.75) | 1.74 (-2.44,5.91) | -1.87 (-9.27,5.54) |
|  | 0.54 (-3.64,4.72) | -3.65 (-9.84,2.53) | -2.97 (-7.75,1.82) | _y_PF | -1.23 (-4.94,2.48) | -4.83 (-12.47,2.80) |
|  | 1.77 (-1.85,5.38) | -2.42 (-7.73,2.88) | -1.74 (-5.91,2.44) | 1.23 (-2.48,4.94) | _y_CER | -3.60 (-10.63,3.43) |
|  | 5.37 (-1.63,12.36) | 1.18 (-7.32,9.68) | 1.87 (-5.54,9.27) | 4.83 (-2.80,12.47) | 3.60 (-3.43,10.63) | _y_ADF |

g.FPG

Supplement Figure 5: Sensitivity analysis of network meta-analysis pooled estimates (mean difference with 95% CI ) excluding studies with high-risk of bias: Weight in kg (A); Fat-free mass in kg(B); Waist circumference in cm (C); Low density lipoprotein-LDL in mg/dl (D); Systolic blood pressure -SBP in mmHg(E); Diastolic blood pressure - DBP in mmHg (F); Fasting plasma glucose – FPG in mg/dl (G). Values in bold indicate a statistically significant effect compared to usual diet.

|  | Treatment | | | | | |
| --- | --- | --- | --- | --- | --- | --- |
|  | Usual diet | mADF | TRE | PF | CER | ADF |
| Comparator | Usual diet | **-7.33 (-9.97,-4.70)** | **-3.95 (-5.61,-2.30)** | **-4.10 (-5.90,-2.31)** | **-3.94 (-5.31,-2.56)** | **-5.38 (-7.63,-3.13)** |
|  | 7.33 (4.70,9.97) | Madf | 3.38 (0.60,6.16) | 3.23 (0.54,5.92) | 3.40 (1.05,5.74) | 1.95 (-1.33,5.23) |
|  | 3.95 (2.30,5.61) | **-3.38 (-6.16,-0.60)** | TRE | -0.15 (-2.16,1.86) | 0.02 (-1.55,1.58) | -1.43 (-4.07,1.21) |
|  | 4.10 (2.31,5.90) | **-3.23 (-5.92,-0.54)** | 0.15 (-1.86,2.16) | PF | 0.17 (-1.17,1.50) | -1.28 (-3.93,1.38) |
|  | 3.94 (2.56,5.31) | **-3.40 (-5.74,-1.05)** | -0.02 (-1.58,1.55) | -0.17 (-1.50,1.17) | CER | -1.44 (-3.80,0.91) |
|  | 5.38 (3.13,7.63) | -1.95 (-5.23,1.33) | 1.43 (-1.21,4.07) | 1.28 (-1.38,3.93) | 1.44 (-0.91,3.80) | ADF |

a.weight

|  | Treatment | | | | | |
| --- | --- | --- | --- | --- | --- | --- |
|  | Usual diet | mADF | TRE | PF | CER | ADF |
| Comparator | Usual diet | **-1.34 (-2.50,-0.18)** | -0.56 (-1.22,0.10) | **-1.41 (-2.27,-0.55)** | **-0.92 (-1.59,-0.24)** | -0.71 (-1.70,0.29) |
|  | 1.34 (0.18,2.50) | mADF | 0.78 (-0.39,1.96) | -0.07 (-1.25,1.10) | 0.42 (-0.57,1.42) | 0.63 (-0.83,2.10) |
|  | 0.56 (-0.10,1.22) | **-0.78 (-1.96,0.39)** | TRE | -0.85 (-1.74,0.03) | -0.36 (-1.01,0.29) | -0.15 (-1.32,1.02) |
|  | 1.41 (0.55,2.27) | 0.07 (-1.10,1.25) | 0.85 (-0.03,1.74) | PF | 0.49 (-0.15,1.13) | 0.70 (-0.54,1.95) |
|  | 0.92 (0.24,1.59) | -0.42 (-1.42,0.57) | 0.36 (-0.29,1.01) | -0.49 (-1.13,0.15) | CER | 0.21 (-0.91,1.33) |
|  | 0.71 (-0.29,1.70) | -0.63 (-2.10,0.83) | 0.15 (-1.02,1.32) | -0.70 (-1.95,0.54) | -0.21 (-1.33,0.91) | ADF |

b.ffm

|  | Treatment | | | | |
| --- | --- | --- | --- | --- | --- |
|  | Usual diet | mADF | TRE | PF | CER |
| Comparator | Usual diet | **-6.58 (-9.54,-3.62)** | **-5.06 (-6.46,-3.65)** | **-3.55 (-5.27,-1.83)** | **-3.33 (-4.67,-1.99)** |
|  | 6.58 (3.62,9.54) | mADF | 1.52 (-1.37,4.42) | 3.03 (0.11,5.96) | 3.25 (0.61,5.89) |
|  | 5.06 (3.65,6.46) | -1.52 (-4.42,1.37) | TRE | 1.51 (-0.30,3.32) | 1.73 (0.53,2.92) |
|  | 3.55 (1.83,5.27) | -3.03 (-5.96,-0.11) | -1.51 (-3.32,0.30) | PF | 0.22 (-1.04,1.47) |
|  | 3.33 (1.99,4.67) | -3.25 (-5.89,-0.61) | -1.73 (-2.92,-0.53) | -0.22 (-1.47,1.04) | CER |

c.wc

|  | Treatment | | | | | |
| --- | --- | --- | --- | --- | --- | --- |
|  | Usual diet | mADF | TRE | PF | CER | ADF |
| Comparator | Usual diet | 7.64 (-5.23,20.52) | -3.86 (-9.72,1.99) | **-8.67 (-15.34,-2.00)** | -4.71 (-9.97,0.54) | 0.50 (-8.89,9.89) |
|  | -7.64 (-20.52,5.23) | Madf | -11.51 (-25.08,2.07) | -16.31 (-29.90,-2.73) | -12.36 (-25.23,0.52) | -7.15 (-22.67,8.38) |
|  | 3.86 (-1.99,9.72) | 11.51 (-2.07,25.08) | TRE | -4.81 (-11.79,2.18) | -0.85 (-6.35,4.65) | 4.36 (-6.01,14.73) |
|  | 8.67 (2.00,15.34) | 16.31 (2.73,29.90) | 4.81 (-2.18,11.79) | PF | 3.96 (-0.59,8.50) | 9.17 (-1.24,19.57) |
|  | 4.71 (-0.54,9.97) | 12.36 (-0.52,25.23) | 0.85 (-4.65,6.35) | -3.96 (-8.50,0.59) | CER | 5.21 (-4.26,14.68) |
|  | -0.50 (-9.89,8.89) | 7.15 (-8.38,22.67) | -4.36 (-14.73,6.01) | -9.17 (-19.57,1.24) | -5.21 (-14.68,4.26) | ADF |

d.LDL

|  | Treatment | | | | | |
| --- | --- | --- | --- | --- | --- | --- |
|  | Usual diet | mADF | TRE | PF | CER | ADF |
| Comparator | Usual diet | -7.91 (-22.52,6.71) | **-5.62 (-7.70,-3.54)** | **-4.99 (-7.28,-2.71)** | **-5.65 (-7.63,-3.67)** | 0.33 (-2.91,3.57) |
|  | 7.91 (-6.71,22.52) | mADF | 2.29 (-12.40,16.98) | 2.91 (-11.81,17.64) | 2.26 (-12.42,16.94) | 8.23 (-6.72,23.19) |
|  | 5.62 (3.54,7.70) | **-2.29 (-16.98,12.40)** | TRE | 0.63 (-0.92,2.17) | -0.03 (-0.75,0.69) | 5.94 (2.34,9.55) |
|  | 4.99 (2.71,7.28) | -2.91 (-17.64,11.81) | -0.63 (-2.17,0.92) | PF | -0.66 (-2.03,0.72) | 5.32 (1.58,9.06) |
|  | 5.65 (3.67,7.63) | -2.26 (-16.94,12.42) | 0.03 (-0.69,0.75) | 0.66 (-0.72,2.03) | CER | 5.97 (2.43,9.51) |
|  | -0.33 (-3.57,2.91) | -8.23 (-23.19,6.72) | -5.94 (-9.55,-2.34) | -5.32 (-9.06,-1.58) | -5.97 (-9.51,-2.43) | ADF |

e.SBP

|  | Treatment | | | | | |
| --- | --- | --- | --- | --- | --- | --- |
|  | Usual diet | mADF | TRE | PF | CER | ADF |
| Comparator | Usual diet | -3.30 (-13.63,7.04) | **-4.26 (-5.36,-3.16)** | **-3.39 (-4.74,-2.04)** | **-3.26 (-4.30,-2.21)** | 1.35 (-0.62,3.31) |
|  | 3.30 (-7.04,13.63) | mADF | -0.97 (-11.33,9.40) | -0.09 (-10.49,10.31) | 0.04 (-10.32,10.40) | 4.64 (-5.87,15.15) |
|  | 4.26 (3.16,5.36) | **0.97 (-9.40,11.33)** | TRE | 0.87 (-0.19,1.94) | 1.00 (0.64,1.37) | 5.61 (3.60,7.62) |
|  | 3.39 (2.04,4.74) | 0.09 (-10.31,10.49) | -0.87 (-1.94,0.19) | PF | 0.13 (-0.87,1.13) | 4.74 (2.55,6.92) |
|  | 3.26 (2.21,4.30) | -0.04 (-10.40,10.32) | -1.00 (-1.37,-0.64) | -0.13 (-1.13,0.87) | CER | 4.61 (2.62,6.59) |
|  | -1.35 (-3.31,0.62) | -4.64 (-15.15,5.87) | -5.61 (-7.62,-3.60) | -4.74 (-6.92,-2.55) | -4.61 (-6.59,-2.62) | ADF |

f.DBP

|  | Treatment | | | | | |
| --- | --- | --- | --- | --- | --- | --- |
|  | Usual diet | mADF | TRE | PF | CER | ADF |
| Comparator | ref | **-4.74 (-9.15,-0.33)** | -1.84 (-4.92,1.23) | -0.74 (-3.77,2.28) | -0.52 (-3.07,2.03) | **-4.81 (-9.01,-0.61)** |
|  | 4.74 (0.33,9.15) | _y_mADF | 2.90 (-2.01,7.80) | 4.00 (-0.24,8.23) | 4.22 (0.46,7.98) | -0.07 (-5.76,5.62) |
|  | 1.84 (-1.23,4.92) | -2.90 (-7.80,2.01) | _y_TRE | 1.10 (-2.52,4.72) | 1.32 (-1.79,4.43) | -2.97 (-7.93,1.99) |
|  | 0.74 (-2.28,3.77) | -4.00 (-8.23,0.24) | -1.10 (-4.72,2.52) | _y_PF | 0.22 (-1.79,2.23) | -4.07 (-8.78,0.65) |
|  | 0.52 (-2.03,3.07) | -4.22 (-7.98,-0.46) | -1.32 (-4.43,1.79) | -0.22 (-2.23,1.79) | _y_CER | -4.29 (-8.65,0.07) |
|  | 4.81 (0.61,9.01) | 0.07 (-5.62,5.76) | 2.97 (-1.99,7.93) | 4.07 (-0.65,8.78) | 4.29 (-0.07,8.65) | _y_ADF |

g.FPG

Supplement Figure 6: Sensitivity analysis of network meta-analysis pooled estimates (mean difference with 95% CI) with medium or long follow up durations (>=3 months). Weight in kg (A); Fat-free mass in kg(B); Waist circumference in cm (C); Low density lipoprotein-LDL in mg/dl (D); Systolic blood pressure -SBP in mmHg(E); Diastolic blood pressure - DBP in mmHg (F); Fasting plasma glucose – FPG in mg/dl (G). Values in bold indicate a statistically significant effect compared to usual diet.
